# Supplementary material for: Mitochondrial-Targeted Therapies Require Mitophagy to Prevent Oxidative Stress Induced by SOD2 Inactivation in Hypertrophied Cardiomyocytes
Source: Antioxidants (Basel). 2022 Apr 6;11(4):723. doi: 10.3390/antiox11040723 (PMC9029275; doi:10.3390/antiox11040723)
Supplement: Supplementary file 1 [file antioxidants-11-00723-s001.zip › antioxidants-1644144-original WB.pdf]

Description: Here, we provided all the uncropped gels corresponding to the western blot showed in the manuscript.

Figure 1D

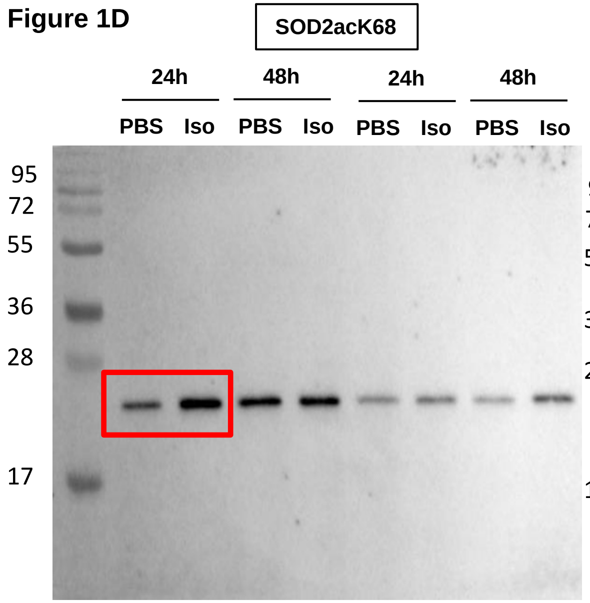
SOD2acK68
24h
48h
24h
48h
PBS Iso PBS Iso PBS Iso PBS Iso
95
72
55
36
28
17

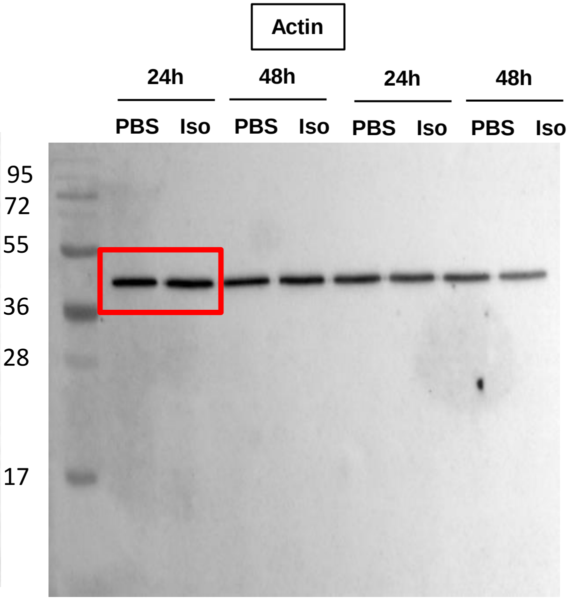
Actin
24h
48h
24h
48h
PBS Iso PBS Iso PBS Iso PBS Iso
95
72
55
36
28
17

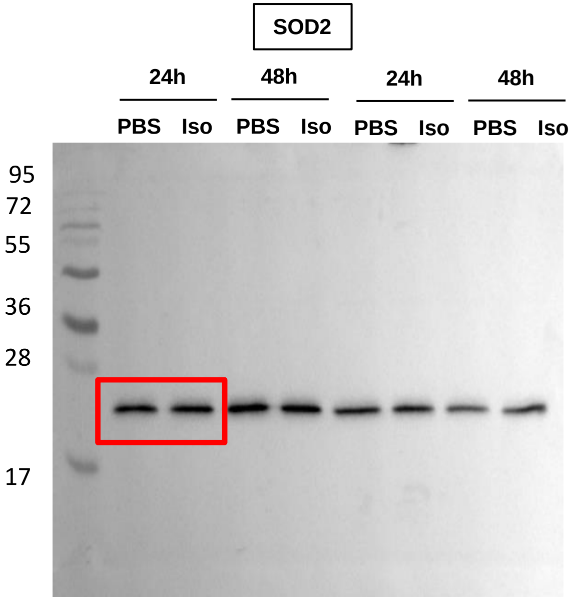
SOD2
24h
48h
24h
48h
PBS Iso PBS Iso PBS Iso PBS Iso
95
72
55
36
28
17

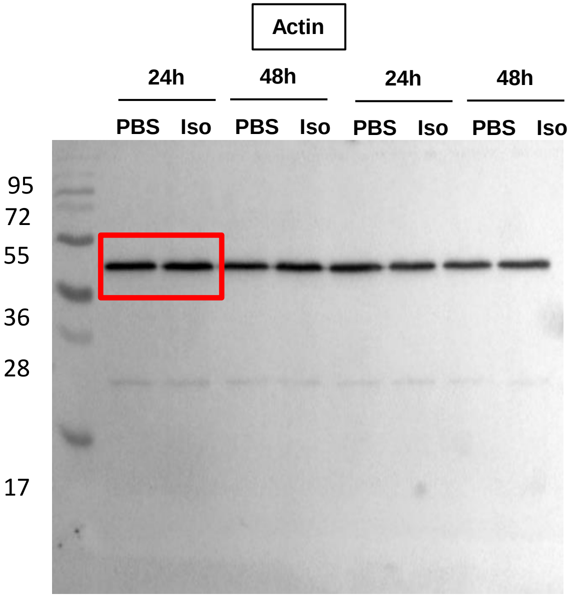
Actin
24h
48h
24h
48h
PBS Iso PBS Iso PBS Iso PBS Iso
95
72
55
36
28
17

Figure 1D

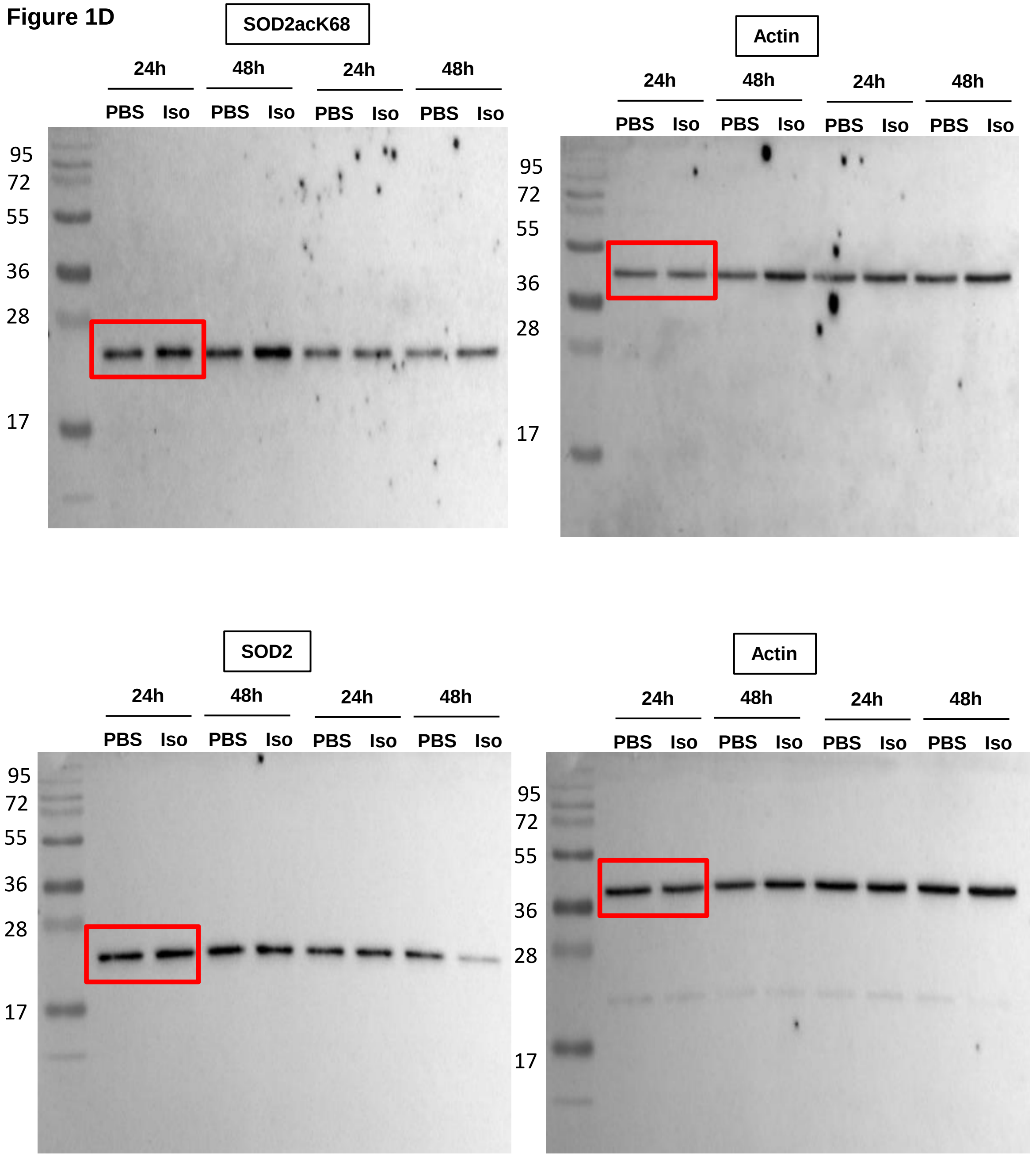
SOD2acK68
24h
48h
24h
48h
PBS
Iso
PBS
Iso
PBS
Iso
PBS
Iso
95
72
55
36
28
17
Actin
24h
48h
24h
48h
PBS
Iso
PBS
Iso
PBS
Iso
PBS
Iso
95
72
55
36
28
17
SOD2
24h
48h
24h
48h
PBS
Iso
PBS
Iso
PBS
Iso
PBS
Iso
95
72
55
36
28
17
Actin
24h
48h
24h
48h
PBS
Iso
PBS
Iso
PBS
Iso
PBS
Iso
95
72
55
36
28
17

Figure 1E

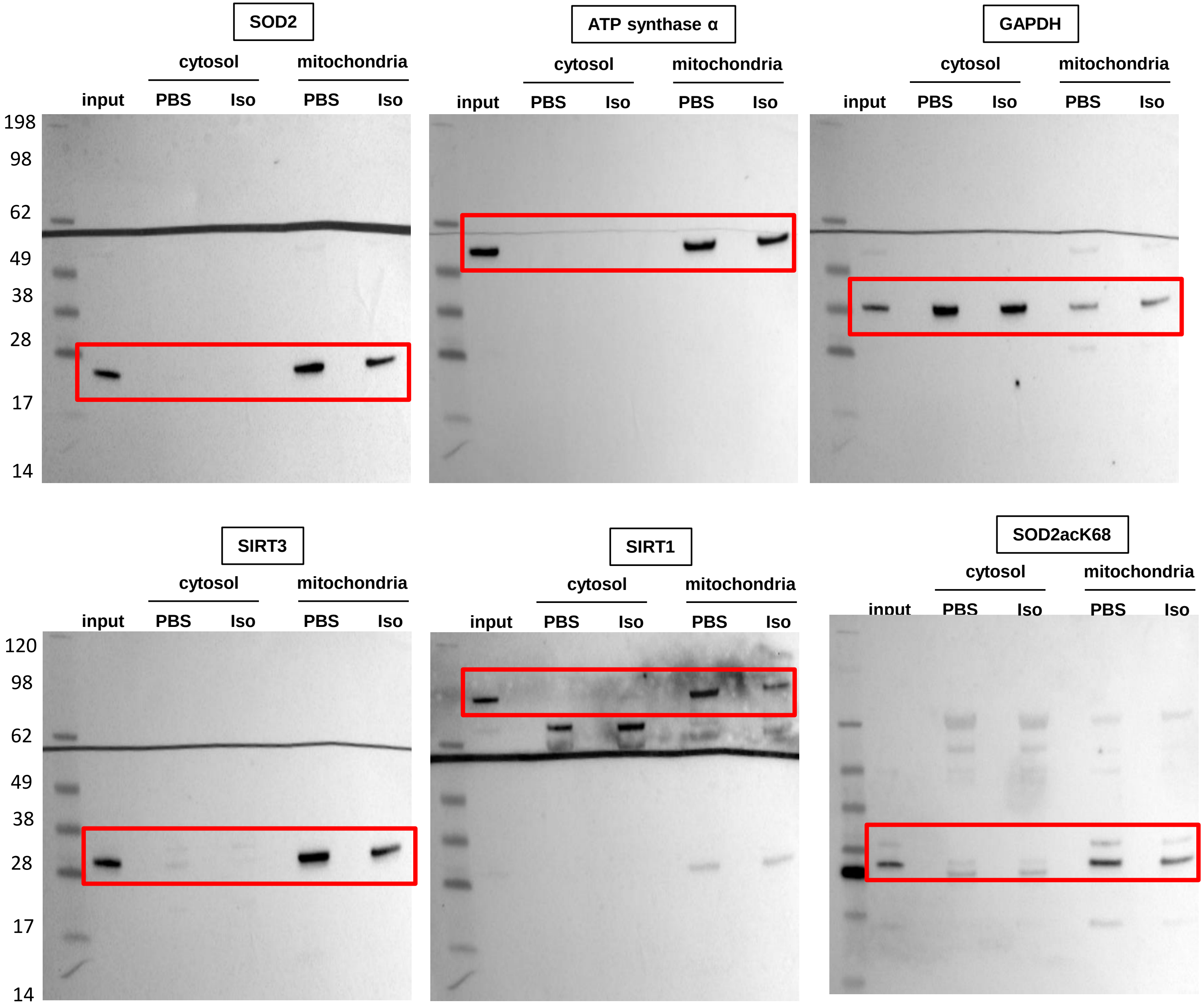
SOD2
cytosol
mitochondria
input
PBS
Iso
PBS
Iso
198
98
62
49
38
28
17
14
ATP synthase α
cytosol
mitochondria
input
PBS
Iso
PBS
Iso
GAPDH
cytosol
mitochondria
input
PBS
Iso
PBS
Iso
SIRT3
cytosol
mitochondria
input
PBS
Iso
PBS
Iso
120
98
62
49
38
28
17
14
SIRT1
cytosol
mitochondria
input
PBS
Iso
PBS
Iso
SOD2acK68
cytosol
mitochondria
input
PBS
Iso
PBS
Iso

Figure S1C

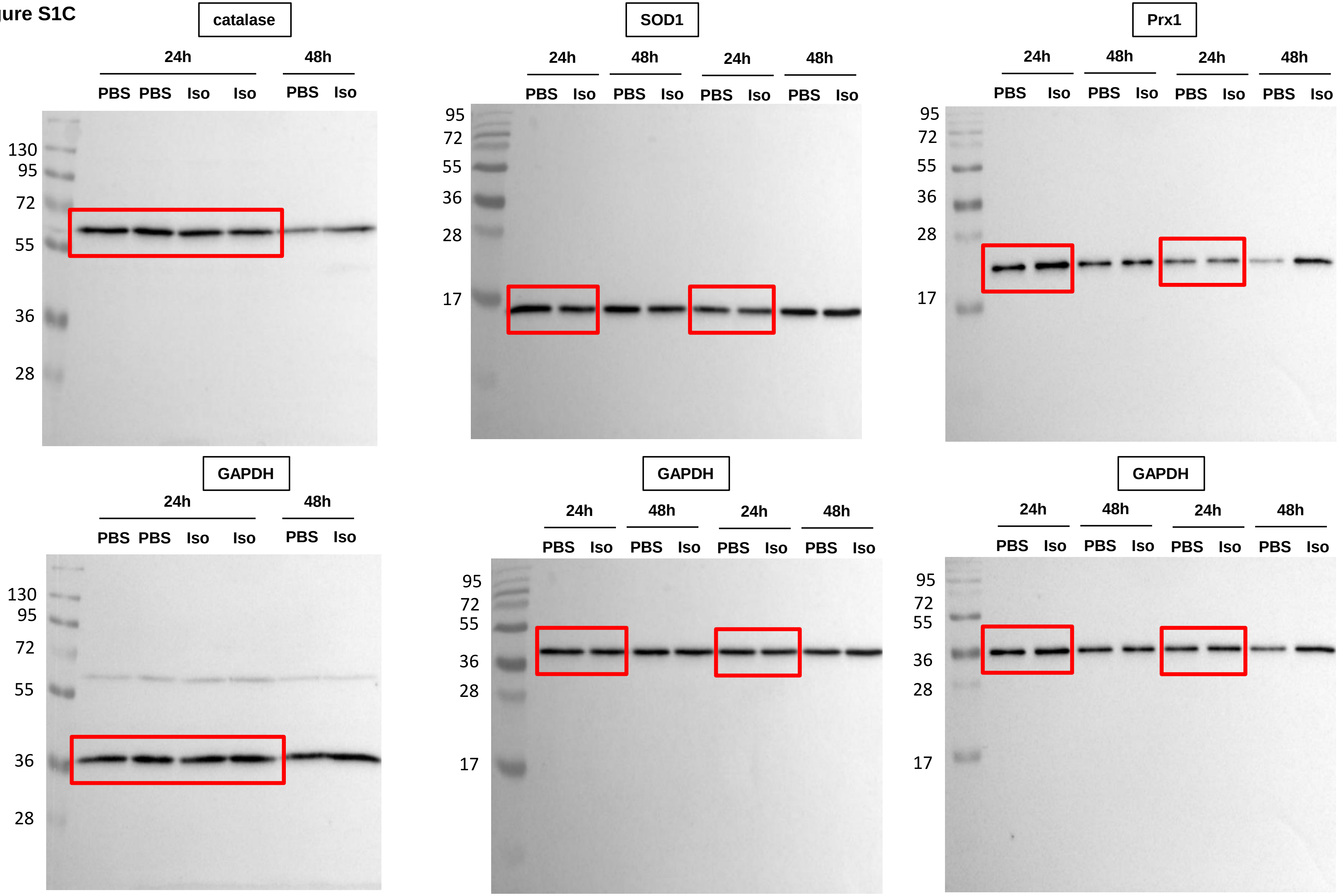
catalase
24h
48h
PBS PBS Iso Iso PBS Iso
130
95
72
55
36
28
SOD1
24h 48h 24h 48h
PBS Iso PBS Iso PBS Iso PBS Iso
95
72
55
36
28
17
Prx1
24h 48h 24h 48h
PBS Iso PBS Iso PBS Iso PBS Iso
95
72
55
36
28
17
GAPDH
24h
48h
PBS PBS Iso Iso PBS Iso
130
95
72
55
36
28
GAPDH
24h 48h 24h 48h
PBS Iso PBS Iso PBS Iso PBS Iso
95
72
55
36
28
17
GAPDH
24h 48h 24h 48h
PBS Iso PBS Iso PBS Iso PBS Iso
95
72
55
36
28
17

Figure S1C

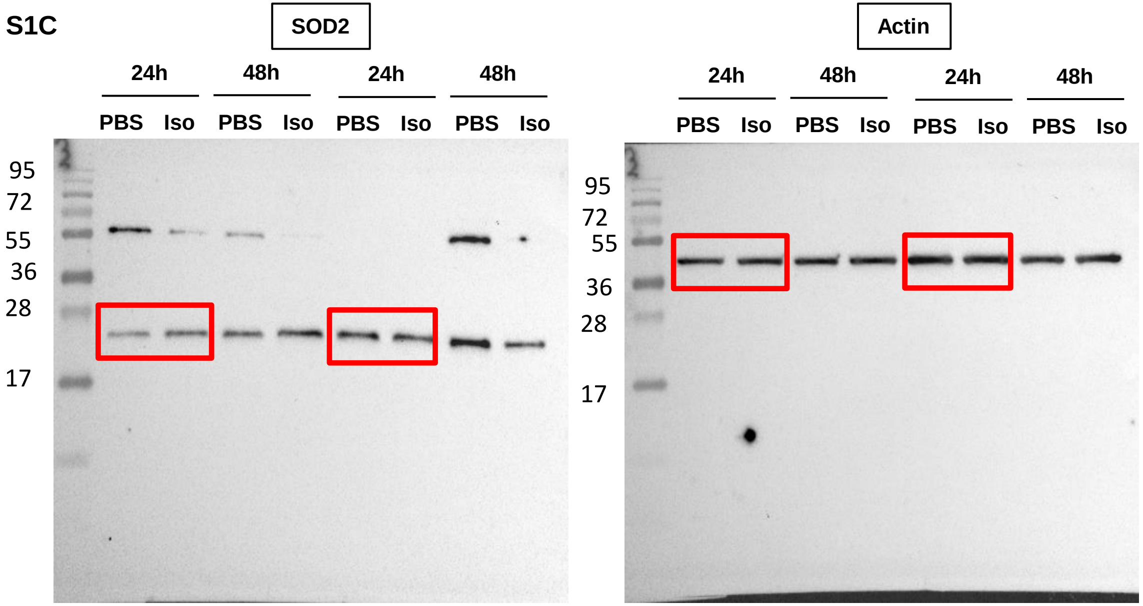
SOD2
24h
48h
24h
48h
PBS
Iso
PBS
Iso
PBS
Iso
PBS
Iso
95
72
55
36
28
17
Actin
24h
48h
24h
48h
PBS
Iso
PBS
Iso
PBS
Iso
PBS
Iso
95
72
55
36
28
17

Figure S1D

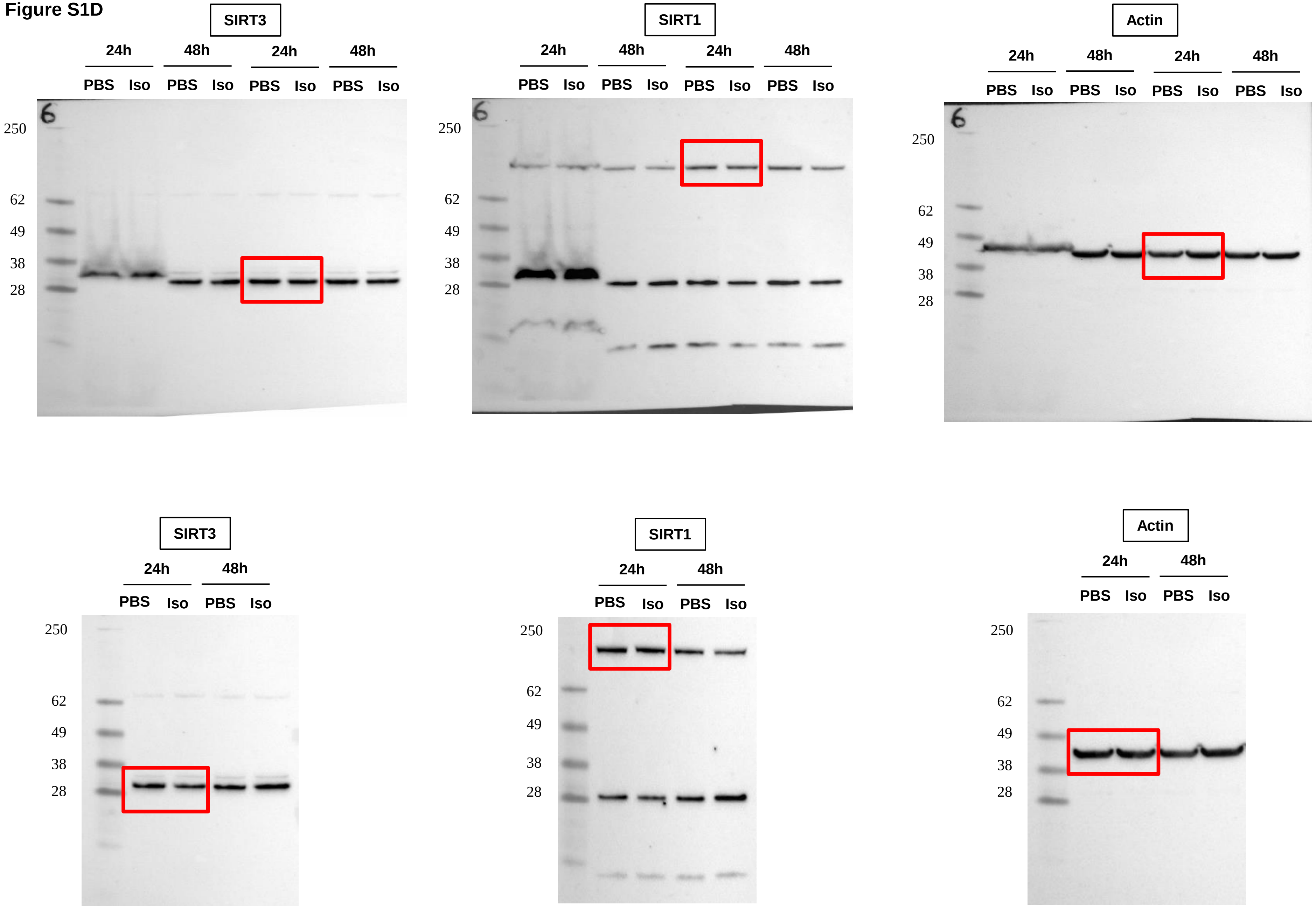
SIRT3
24h
48h
24h
48h
PBS Iso PBS Iso PBS Iso PBS Iso
250
62
49
38
28
SIRT1
24h
48h
24h
48h
PBS Iso PBS Iso PBS Iso PBS Iso
250
62
49
38
28
Actin
24h
48h
24h
48h
PBS Iso PBS Iso PBS Iso PBS Iso
250
62
49
38
28
SIRT3
24h
48h
PBS Iso PBS Iso
250
62
49
38
28
SIRT1
24h
48h
PBS Iso PBS Iso
250
62
49
38
28
Actin
24h
48h
PBS Iso PBS Iso
250
62
49
38
28

Figure S1D

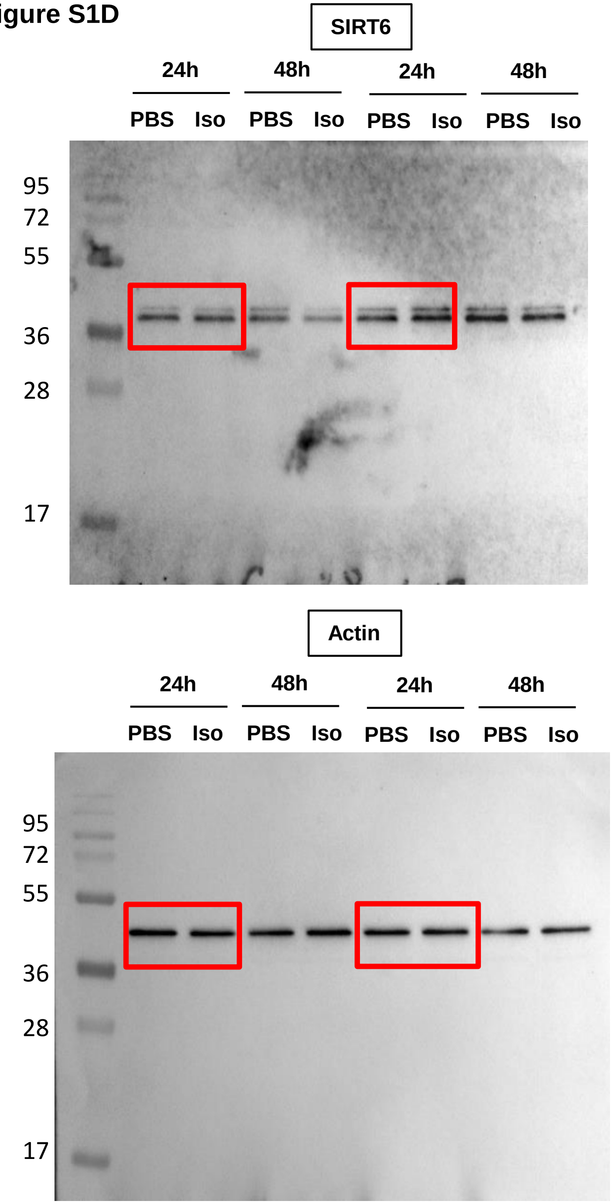
SIRT6
24h
48h
24h
48h
PBS
Iso
PBS
Iso
PBS
Iso
PBS
Iso
95
72
55
36
28
17
Actin
24h
48h
24h
48h
PBS
Iso
PBS
Iso
PBS
Iso
PBS
Iso
95
72
55
36
28
17

Figure S1E

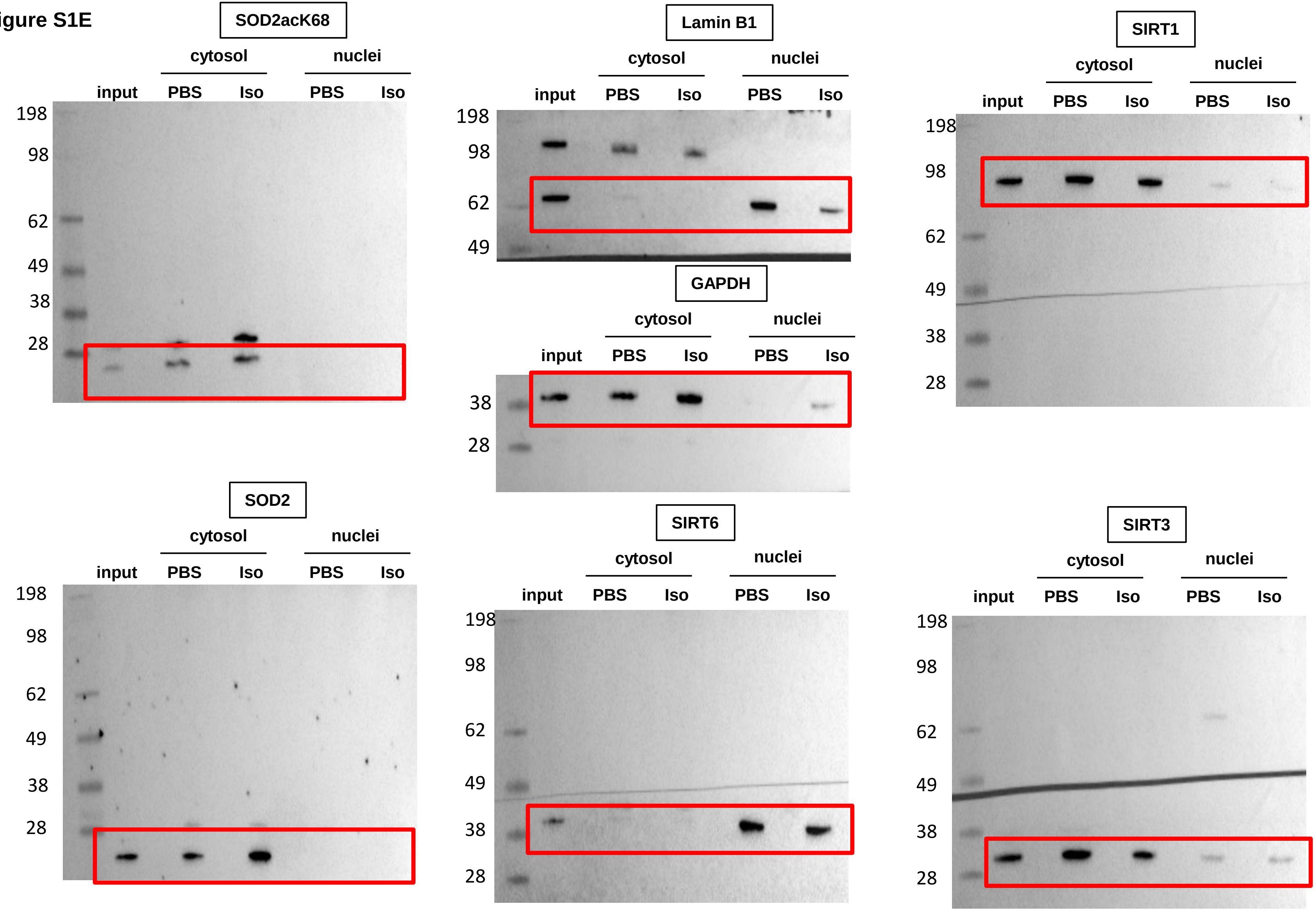
SOD2acK68
cytosol
nuclei
input
PBS
Iso
PBS
Iso
198
98
62
49
38
28
Lamin B1
cytosol
nuclei
input
PBS
Iso
PBS
Iso
198
98
62
49
GAPDH
cytosol
nuclei
input
PBS
Iso
PBS
Iso
38
28
SIRT1
cytosol
nuclei
input
PBS
Iso
PBS
Iso
198
98
62
49
38
28
SOD2
cytosol
nuclei
input
PBS
Iso
PBS
Iso
198
98
62
49
38
28
SIRT6
cytosol
nuclei
input
PBS
Iso
PBS
Iso
198
98
62
49
38
28
SIRT3
cytosol
nuclei
input
PBS
Iso
PBS
Iso
198
98
62
49
38
28

Figure 2A

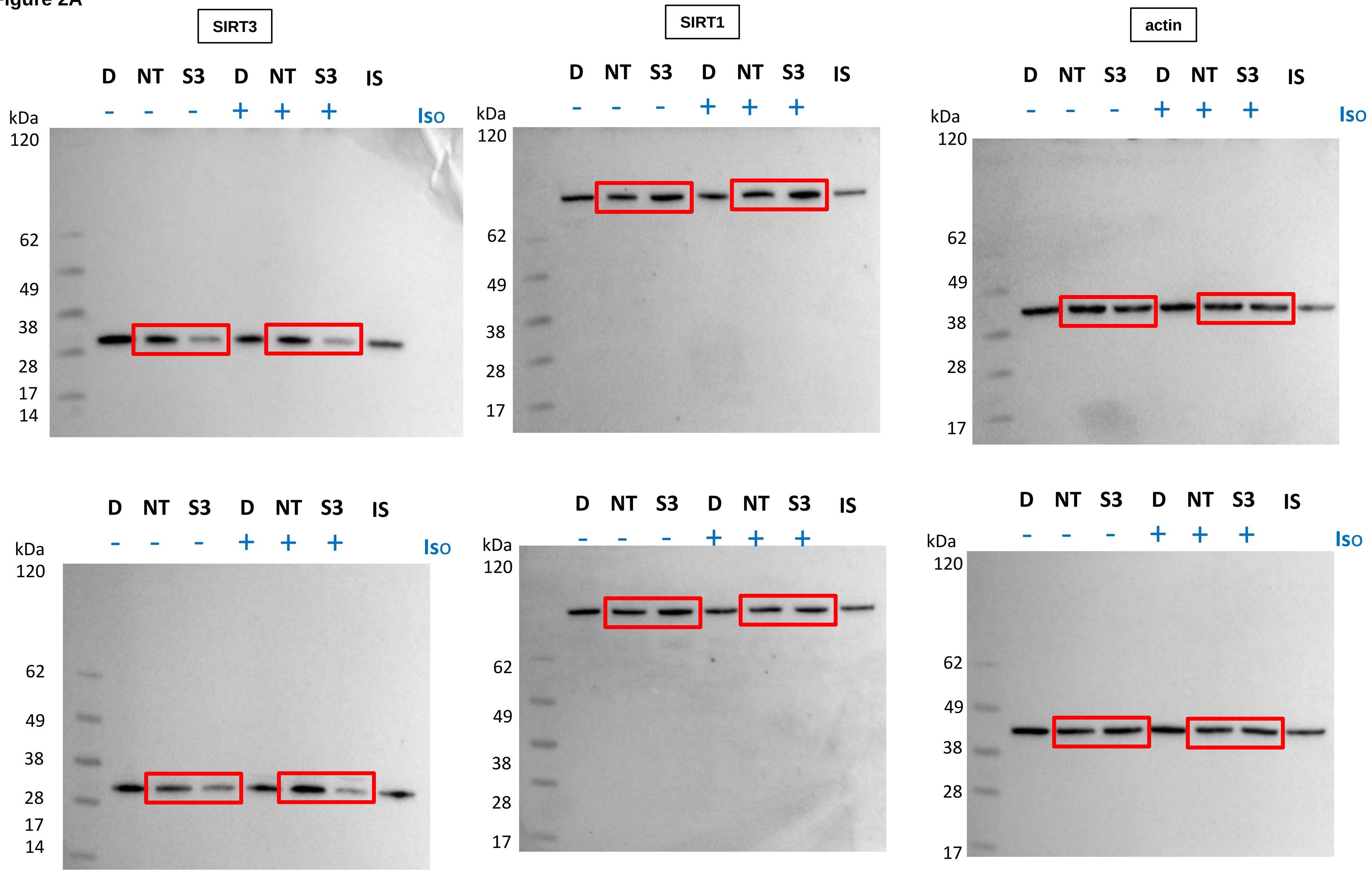

Figure 2B

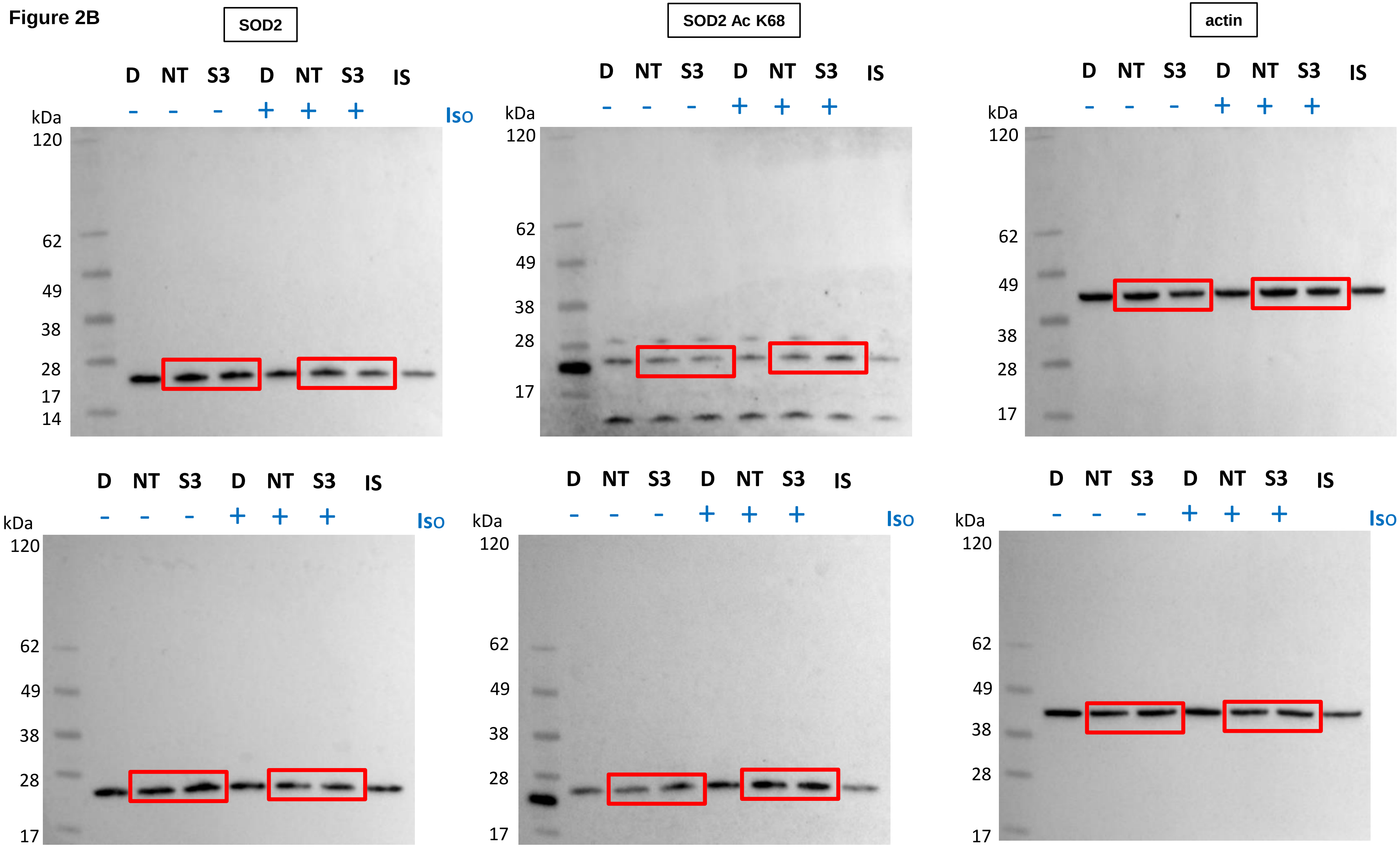
SOD2
SOD2 Ac K68
actin
D NT S3 D NT S3 IS
- - - + + +
Iso
kDa
120
62
49
38
28
17
14

Figure 3A

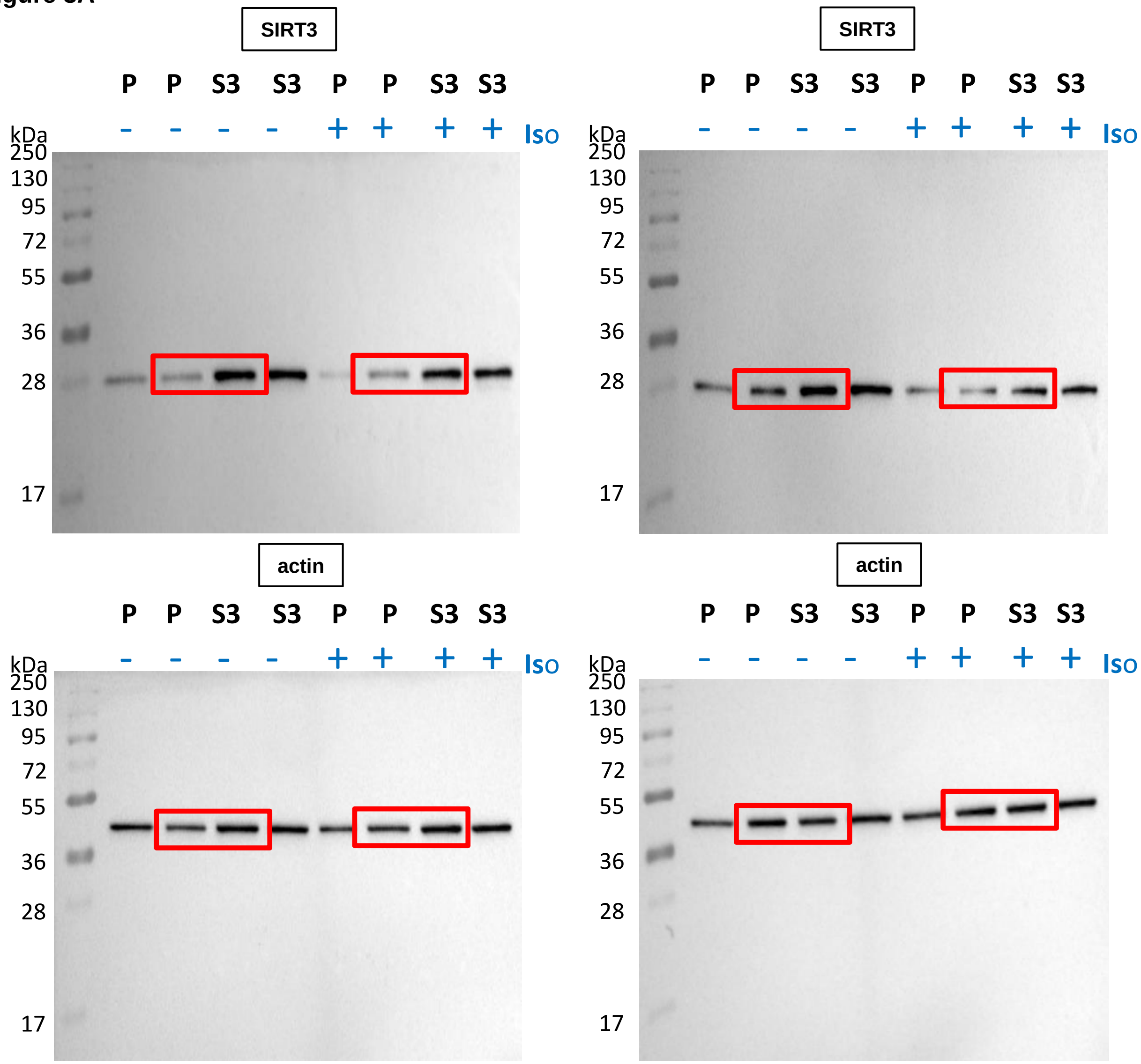
SIRT3
P P S3 S3 P P S3 S3
- - - - + + + + Iso
kDa
250
130
95
72
55
36
28
17
SIRT3
P P S3 S3 P P S3 S3
- - - - + + + + Iso
kDa
250
130
95
72
55
36
28
17
actin
P P S3 S3 P P S3 S3
- - - - + + + + Iso
kDa
250
130
95
72
55
36
28
17
actin
P P S3 S3 P P S3 S3
- - - - + + + + Iso
kDa
250
130
95
72
55
36
28
17

Figure 3A

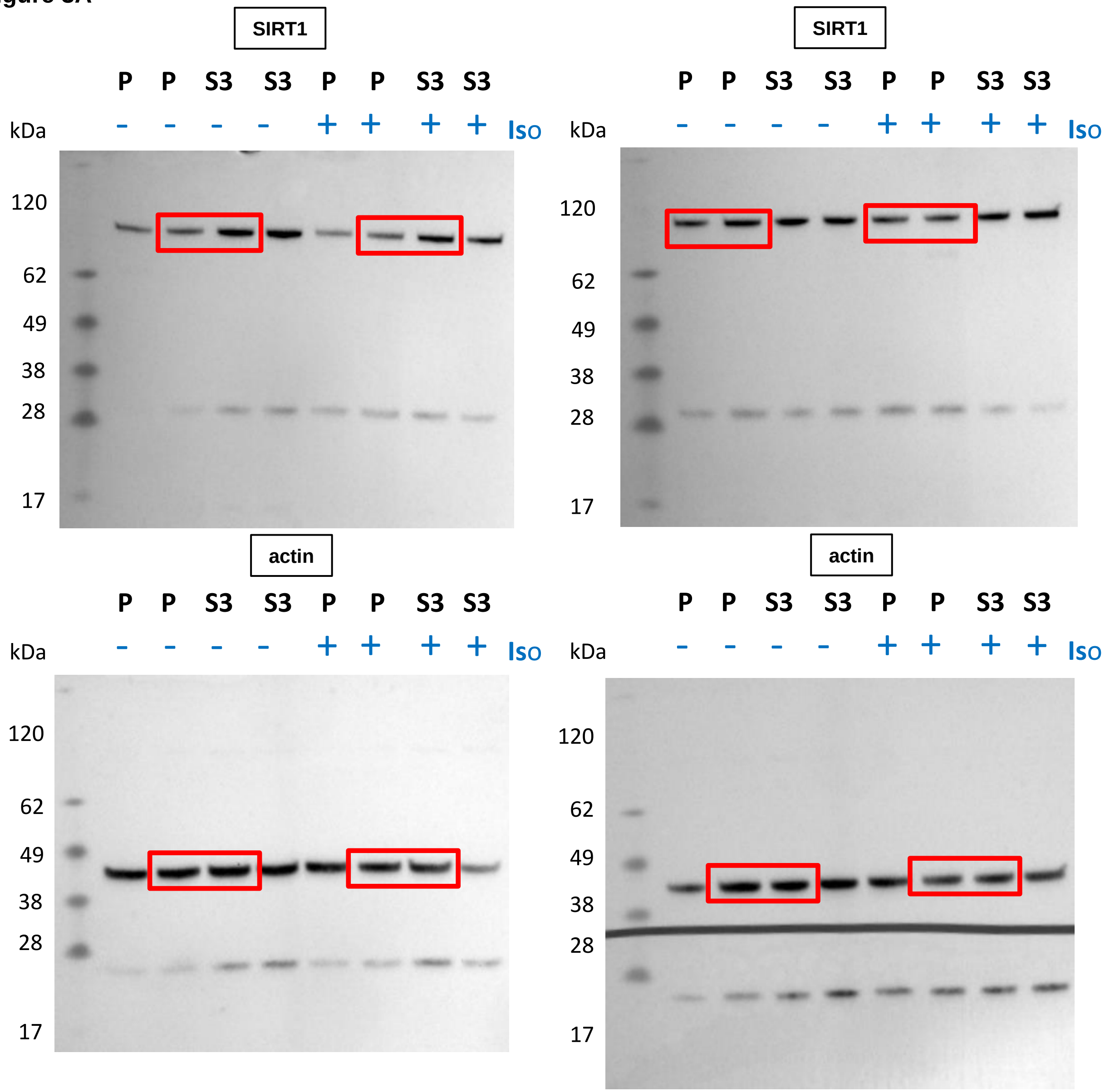
SIRT1
P P S3 S3 P P S3 S3
kDa - - - - + + + + Iso
120
62
49
38
28
17
SIRT1
P P S3 S3 P P S3 S3
kDa - - - - + + + + Iso
120
62
49
38
28
17
actin
P P S3 S3 P P S3 S3
kDa - - - - + + + + Iso
120
62
49
38
28
17
actin
P P S3 S3 P P S3 S3
kDa - - - - + + + + Iso
120
62
49
38
28
17

Figure 3B

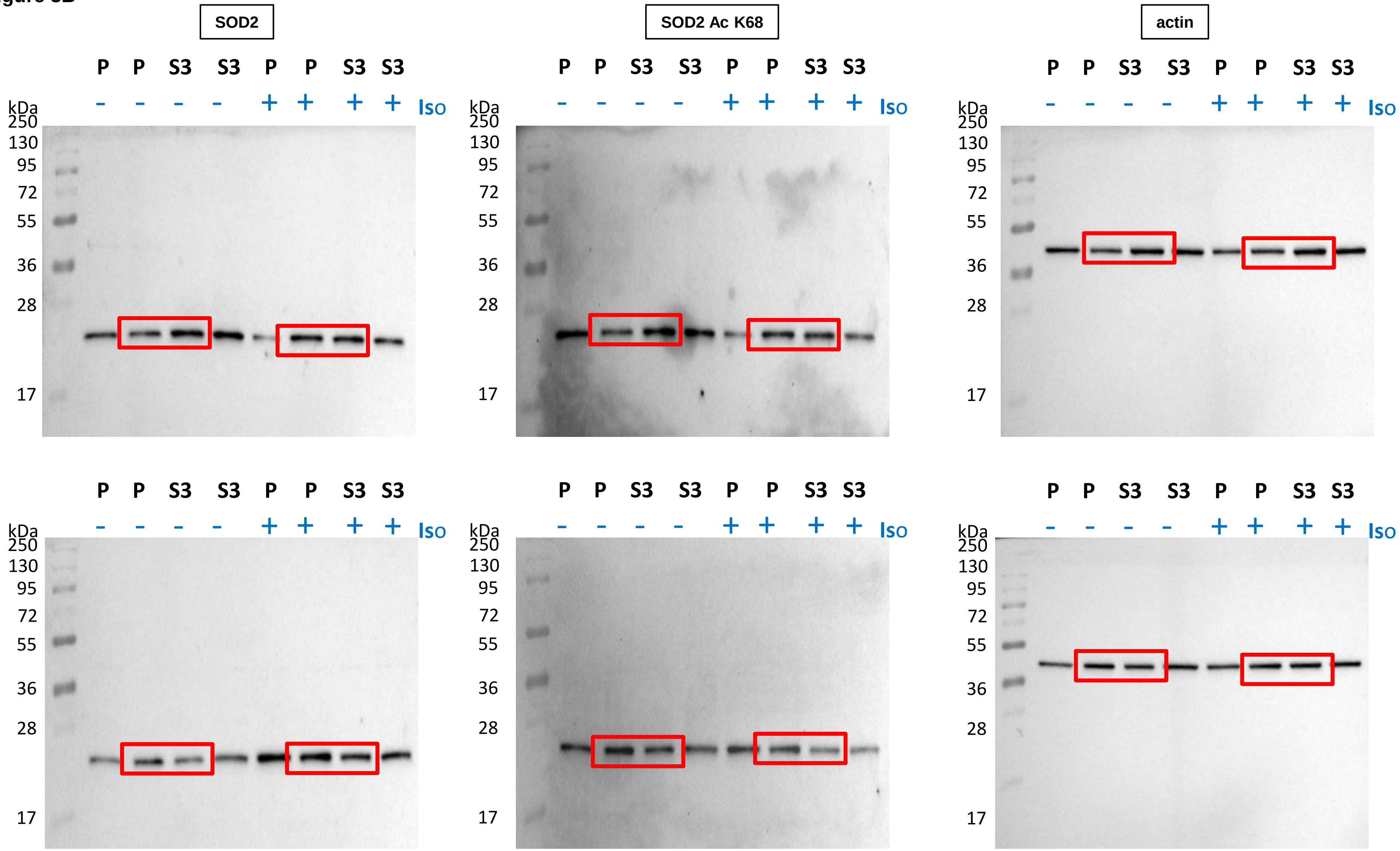

SOD2
SOD2 Ac K68
actin
P P S3 S3 P P S3 S3
- - - - + + + + Iso
kDa
250
130
95
72
55
36
28
17

Figure S2A

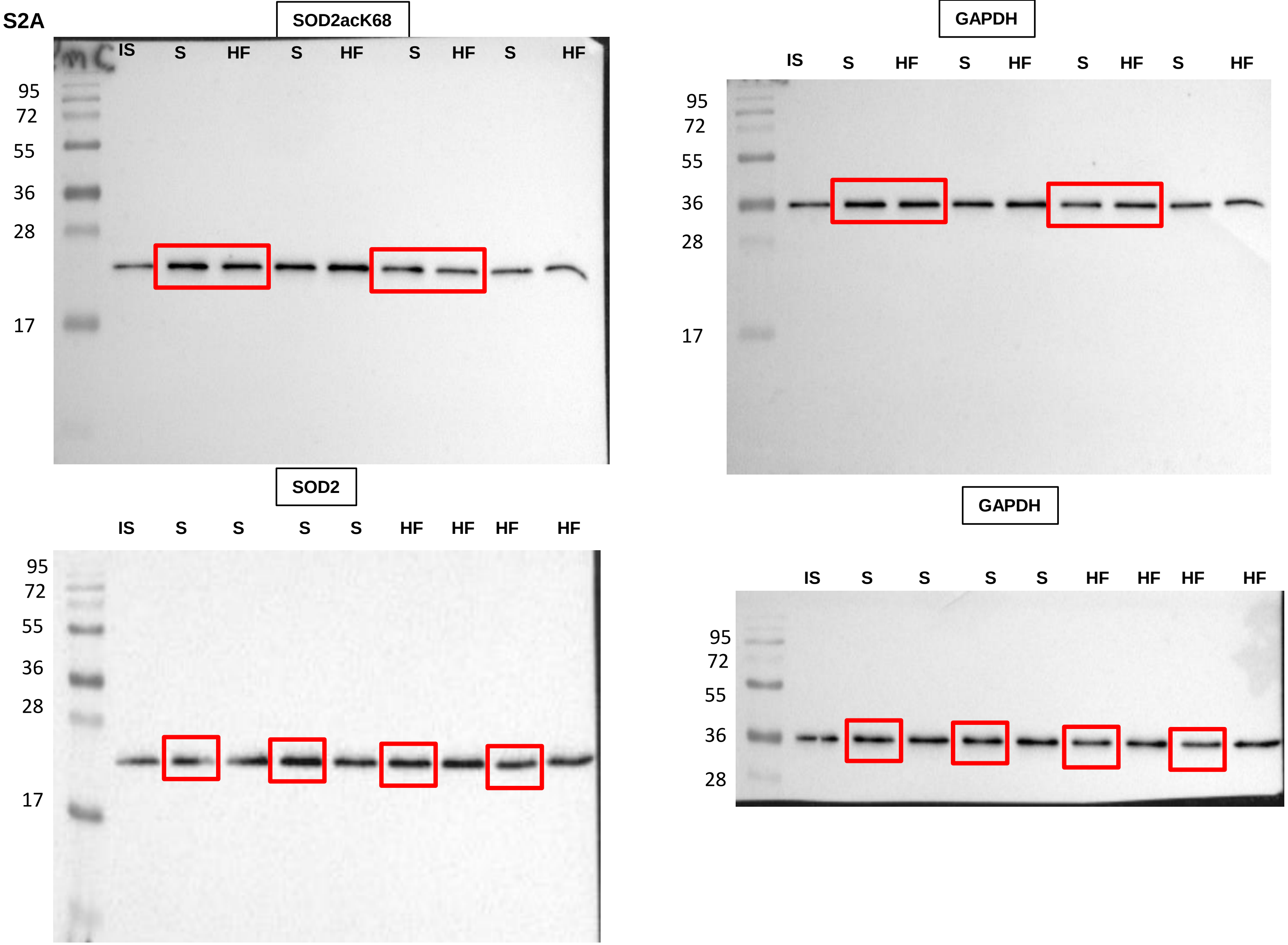

SOD2acK68
IS S HF S HF S HF S HF
95
72
55
36
28
17
GAPDH
IS S HF S HF S HF S HF
95
72
55
36
28
17
SOD2
IS S S S S HF HF HF HF
95
72
55
36
28
17
GAPDH
IS S S S S HF HF HF HF
95
72
55
36
28

Figure S2A

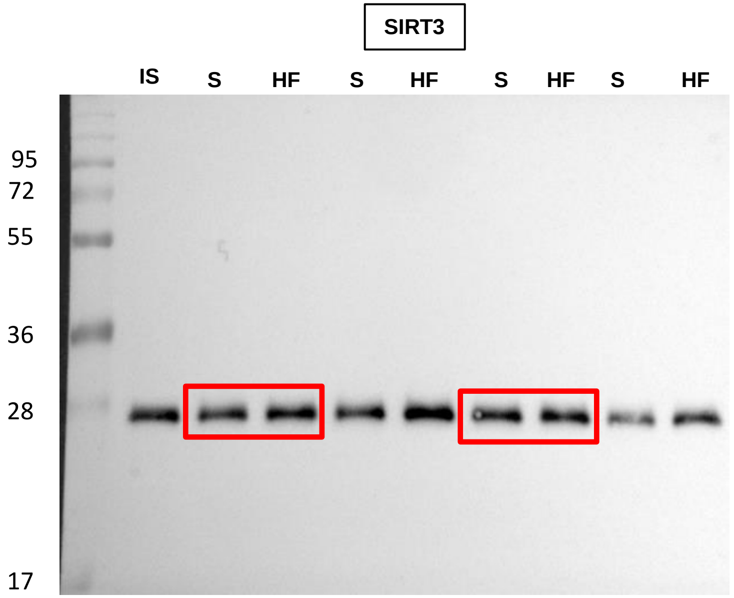
SIRT3
IS S HF S HF S HF S HF
95
72
55
36
28
17

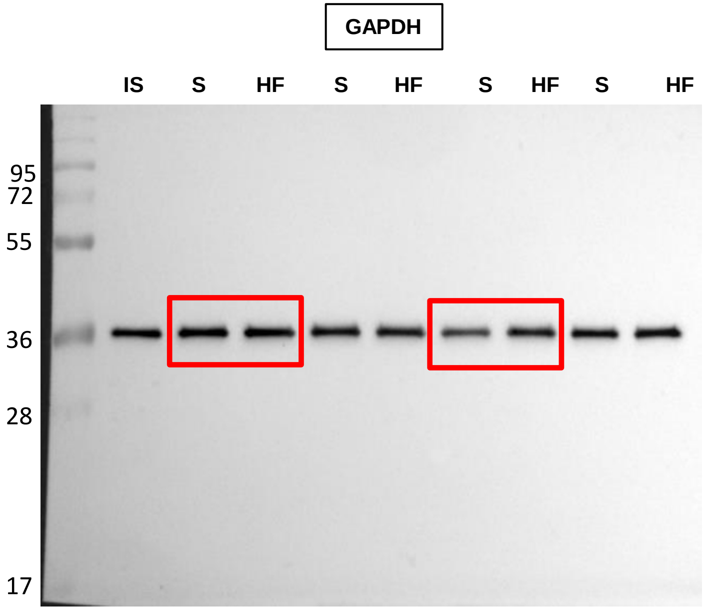
GAPDH
IS S HF S HF S HF S HF
95
72
55
36
28
17

Figure S2B

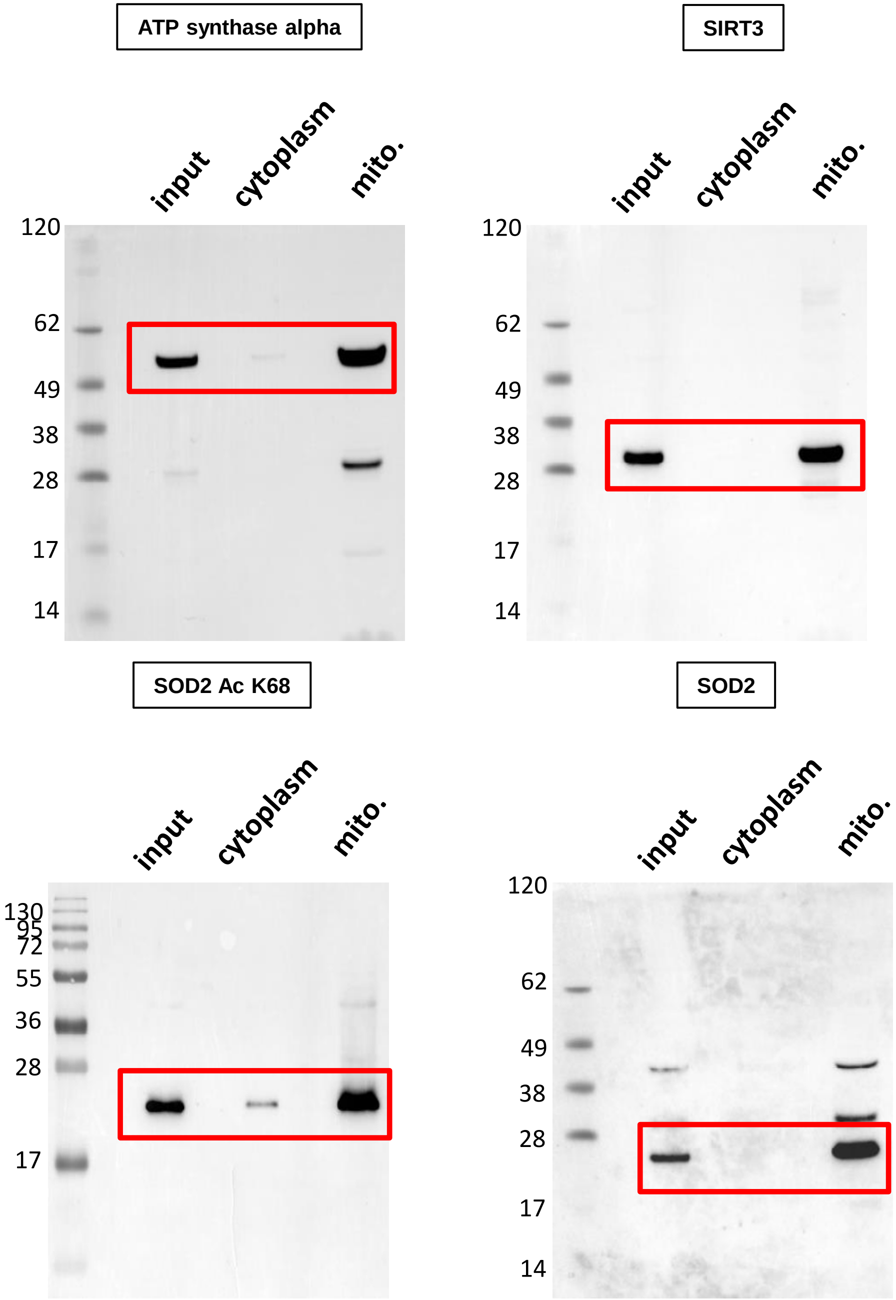

Figure S2C

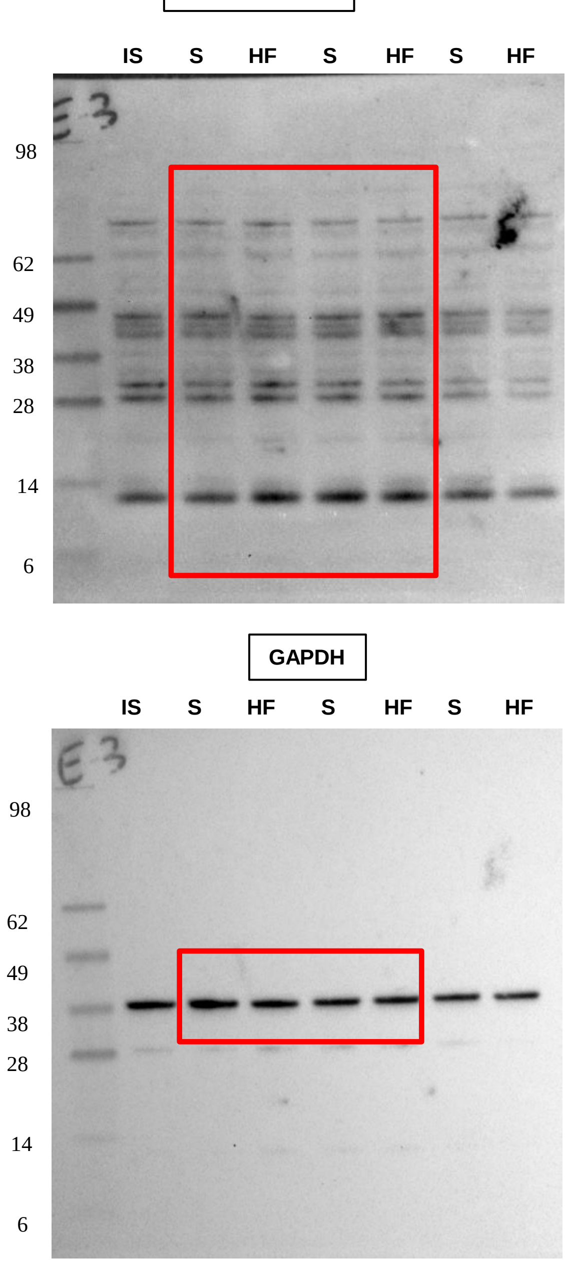

Figure S2D

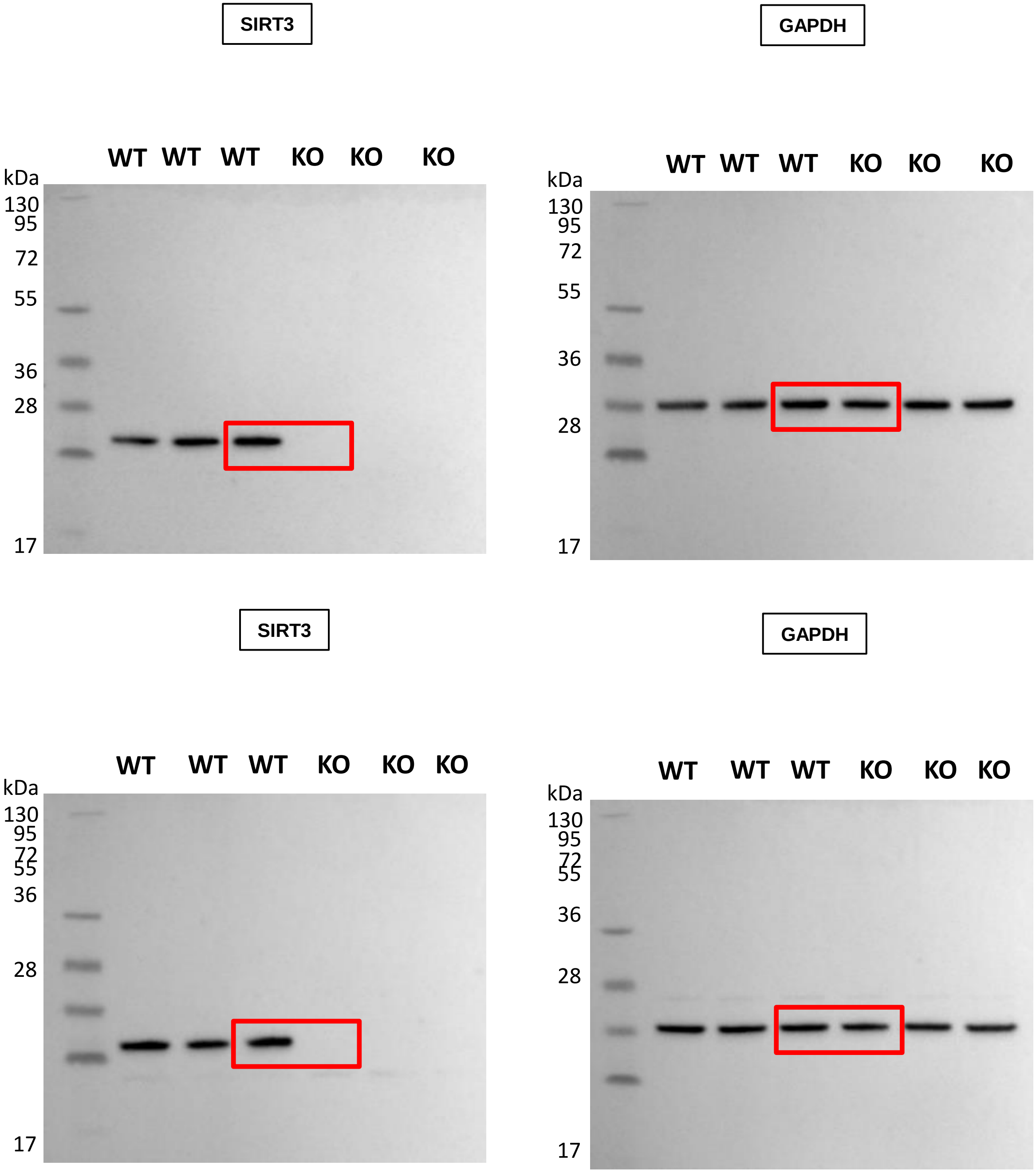
SIRT3
GAPDH
WT WT WT KO KO KO
kDa
130
95
72
55
36
28
17
SIRT3
GAPDH
WT WT WT KO KO KO
kDa
130
95
72
55
36
28
17

Figure S2D

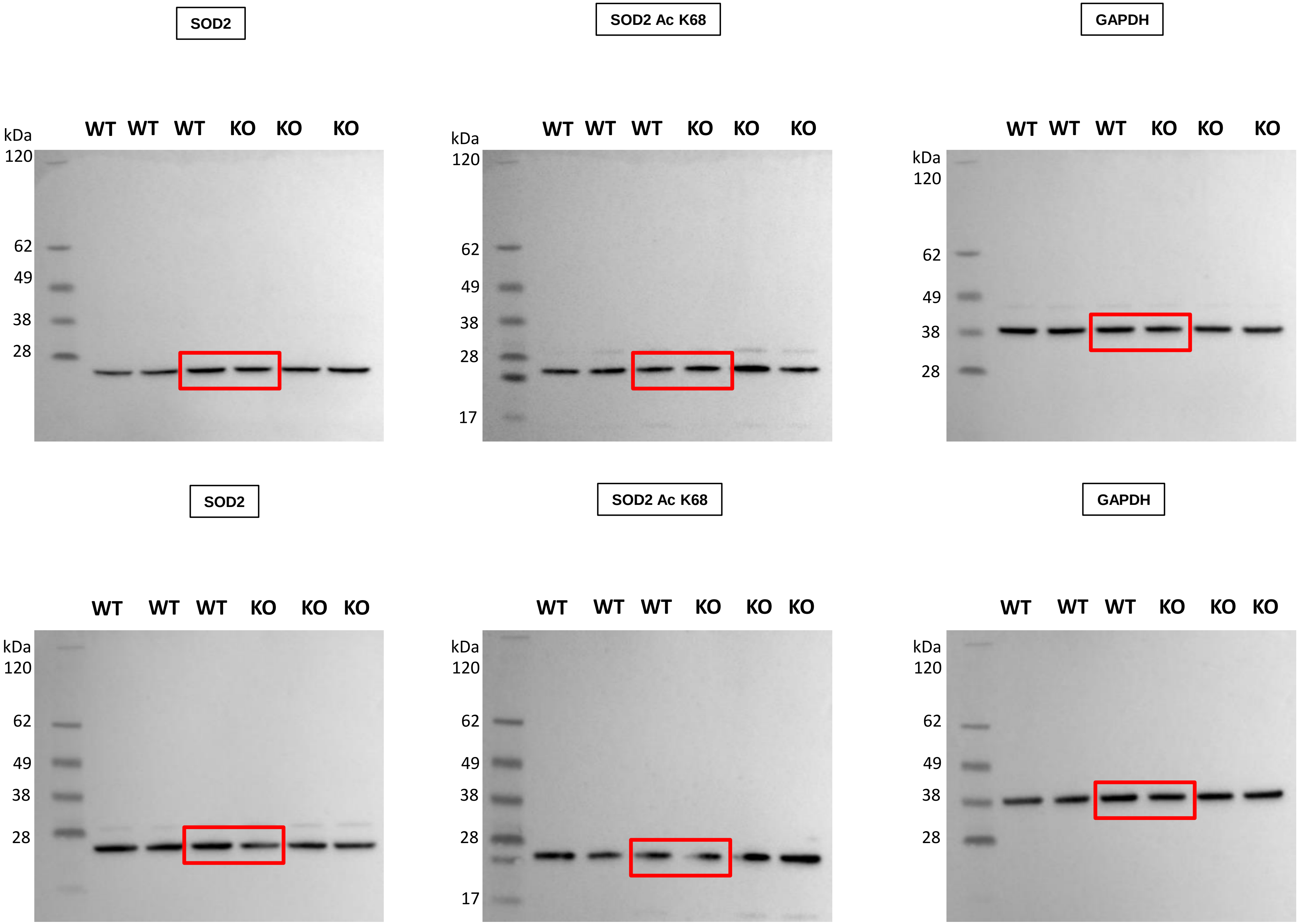
SOD2
SOD2 Ac K68
GAPDH
WT WT WT KO KO KO
kDa
120
62
49
38
28
17
SOD2
SOD2 Ac K68
GAPDH
WT WT WT KO KO KO
kDa
120
62
49
38
28
17

Figure 4c

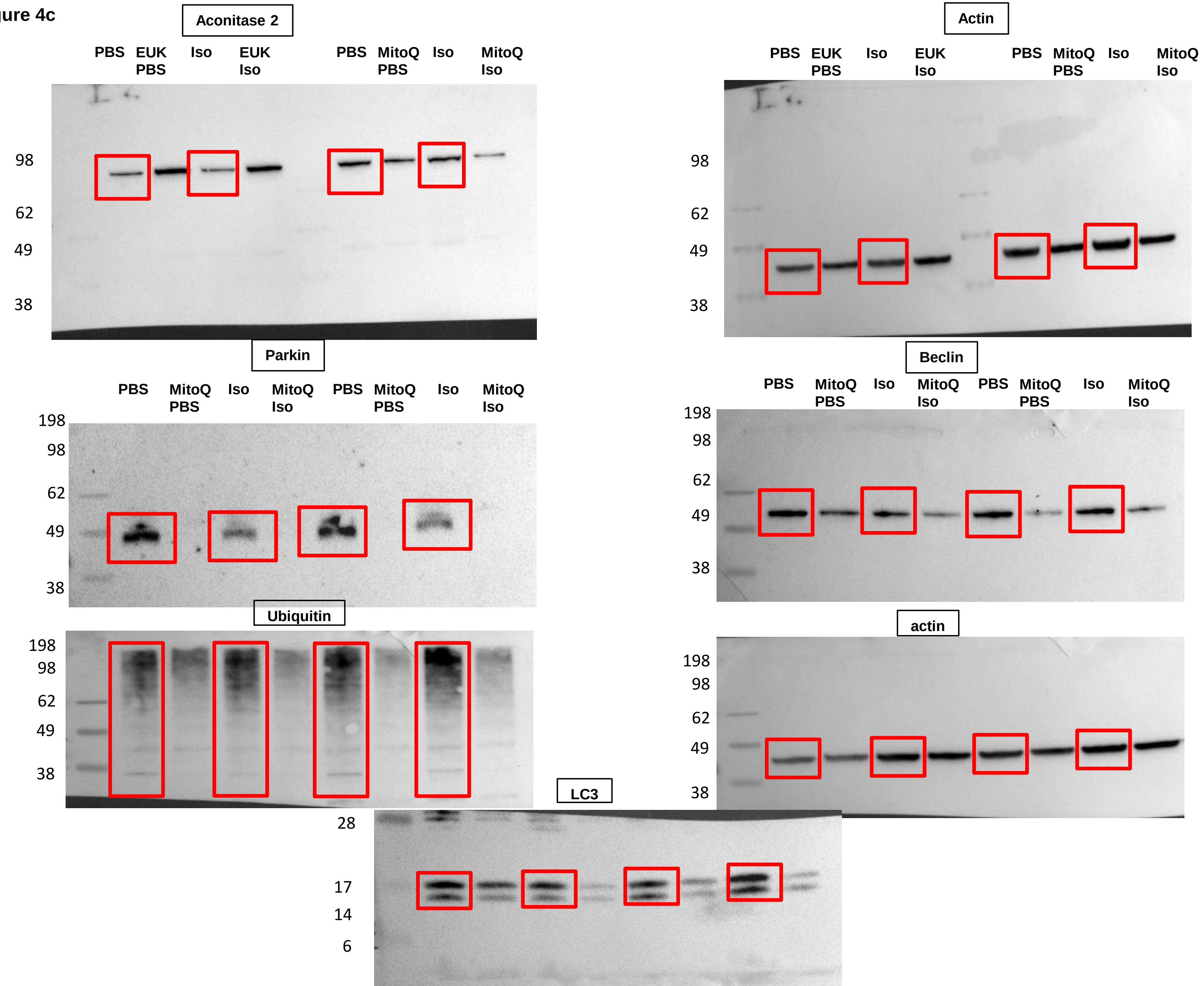
Aconitase 2
PBS
EUK PBS
Iso
EUK Iso
PBS
MitoQ PBS
Iso
MitoQ Iso
98
62
49
38
Actin
PBS
EUK PBS
Iso
EUK Iso
PBS
MitoQ PBS
Iso
MitoQ Iso
98
62
49
38
Parkin
PBS
MitoQ PBS
Iso
MitoQ Iso
PBS
MitoQ PBS
Iso
MitoQ Iso
198
98
62
49
38
Beclin
PBS
MitoQ PBS
Iso
MitoQ Iso
PBS
MitoQ PBS
Iso
MitoQ Iso
198
98
62
49
38
Ubiquitin
198
98
62
49
38
actin
198
98
62
49
38
LC3
28
17
14
6

Figure S3B

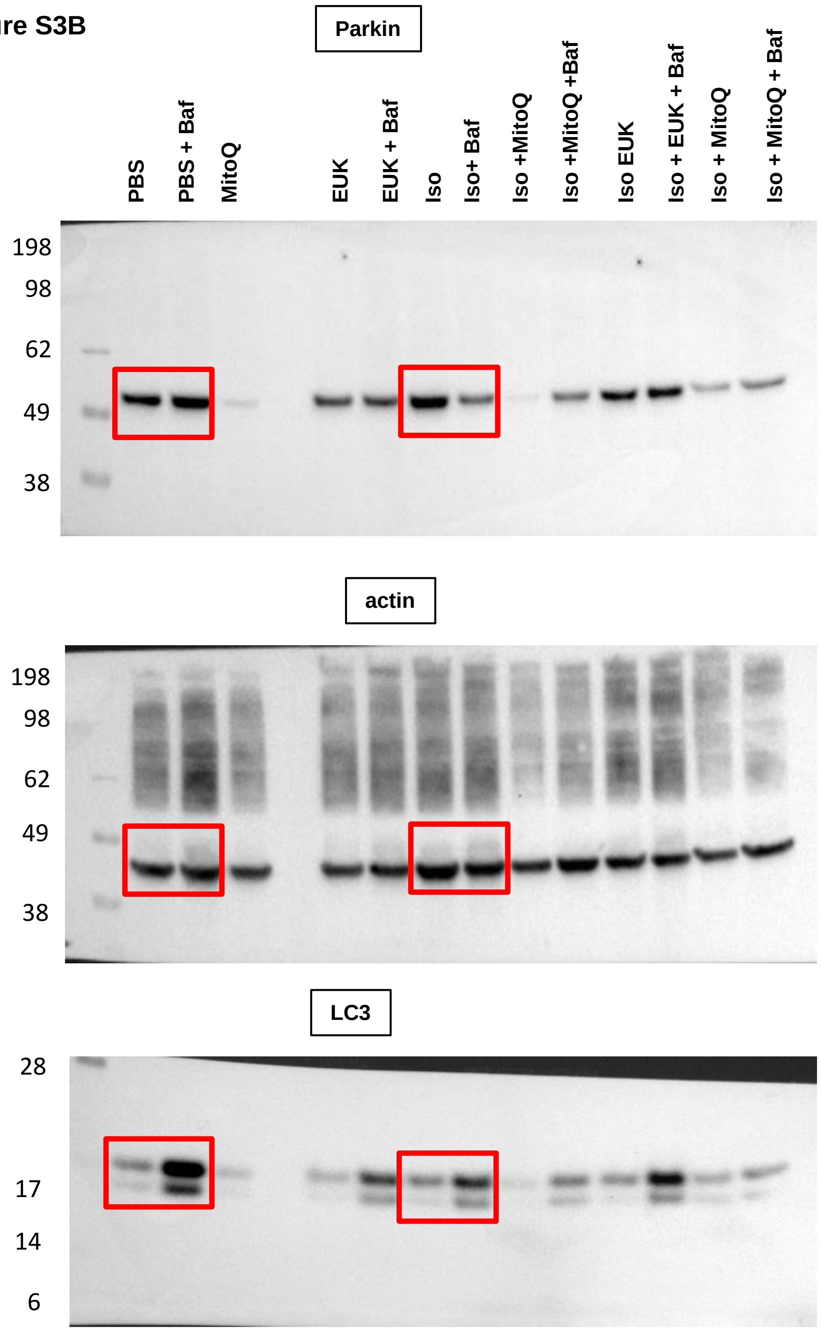

Parkin
PBS
PBS + Baf
MitoQ
EUK
EUK + Baf
Iso
Iso+ Baf
Iso +MitoQ
Iso +MitoQ +Baf
Iso EUK
Iso + EUK + Baf
Iso + MitoQ
Iso + MitoQ + Baf
198
98
62
49
38
actin
198
98
62
49
38
LC3
28
17
14
6

Figure 5D

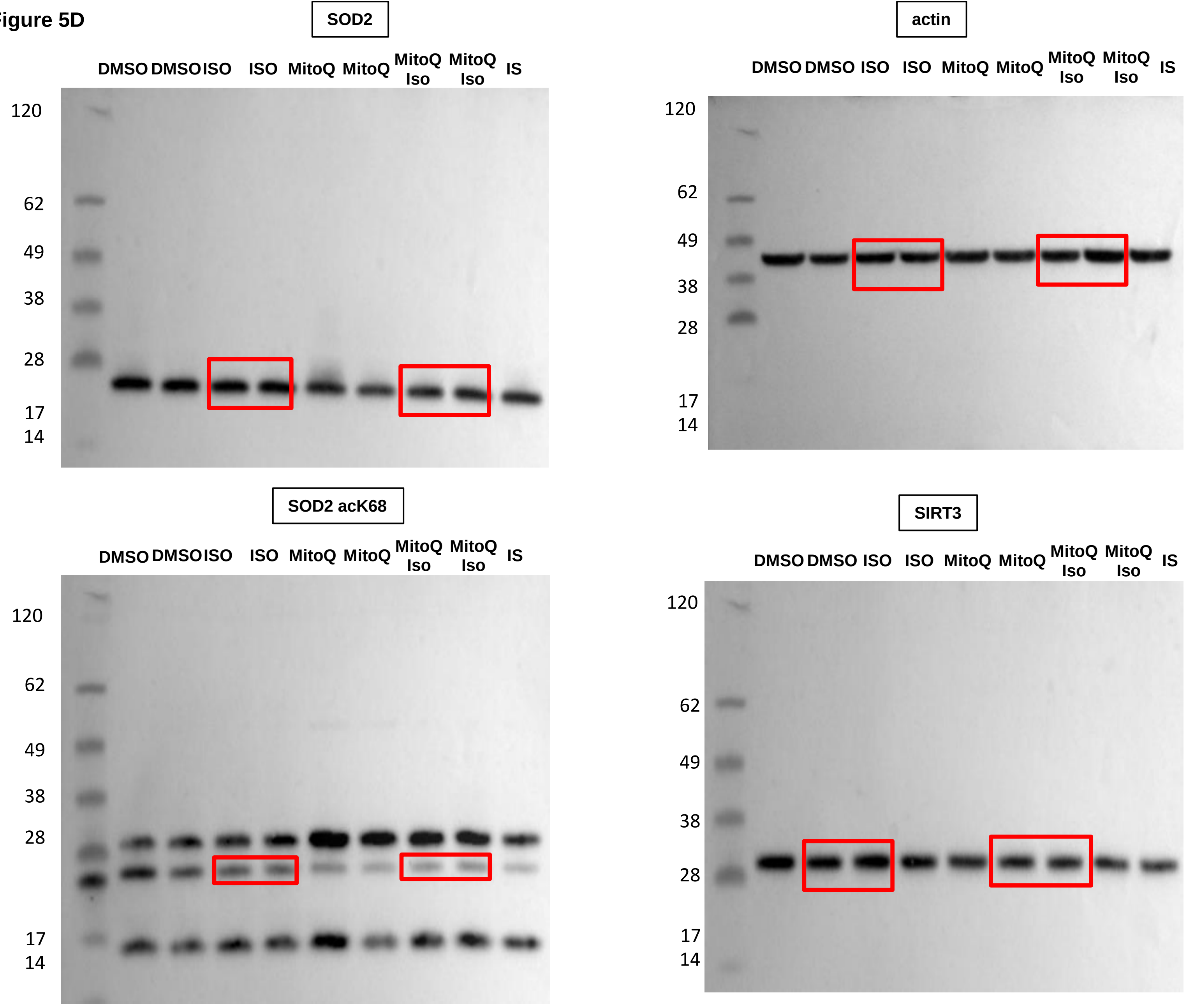
SOD2
DMSO DMSO ISO ISO MitoQ MitoQ MitoQ Iso MitoQ Iso IS
120
62
49
38
28
17
14
actin
DMSO DMSO ISO ISO MitoQ MitoQ MitoQ Iso MitoQ Iso IS
120
62
49
38
28
17
14
SOD2 acK68
DMSO DMSO ISO ISO MitoQ MitoQ MitoQ Iso MitoQ Iso IS
120
62
49
38
28
17
14
SIRT3
DMSO DMSO ISO ISO MitoQ MitoQ MitoQ Iso MitoQ Iso IS
120
62
49
38
28
17
14

Figure S4C

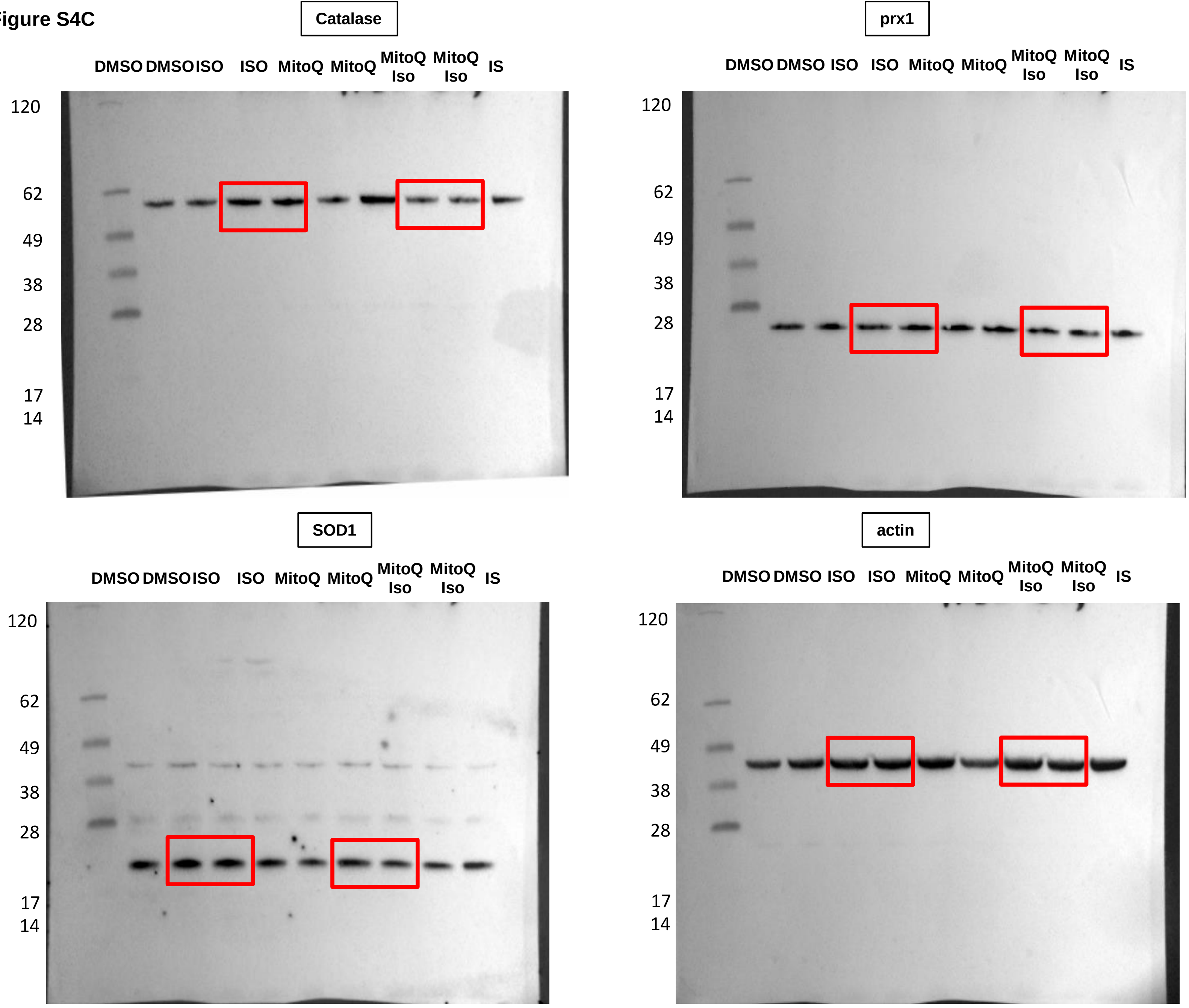
Catalase
DMSO DMSO ISO ISO MitoQ MitoQ MitoQ Iso MitoQ Iso IS
120
62
49
38
28
17
14
prx1
DMSO DMSO ISO ISO MitoQ MitoQ MitoQ Iso MitoQ Iso IS
120
62
49
38
28
17
14
SOD1
DMSO DMSO ISO ISO MitoQ MitoQ MitoQ Iso MitoQ Iso IS
120
62
49
38
28
17
14
actin
DMSO DMSO ISO ISO MitoQ MitoQ MitoQ Iso MitoQ Iso IS
120
62
49
38
28
17
14

Figure S4D

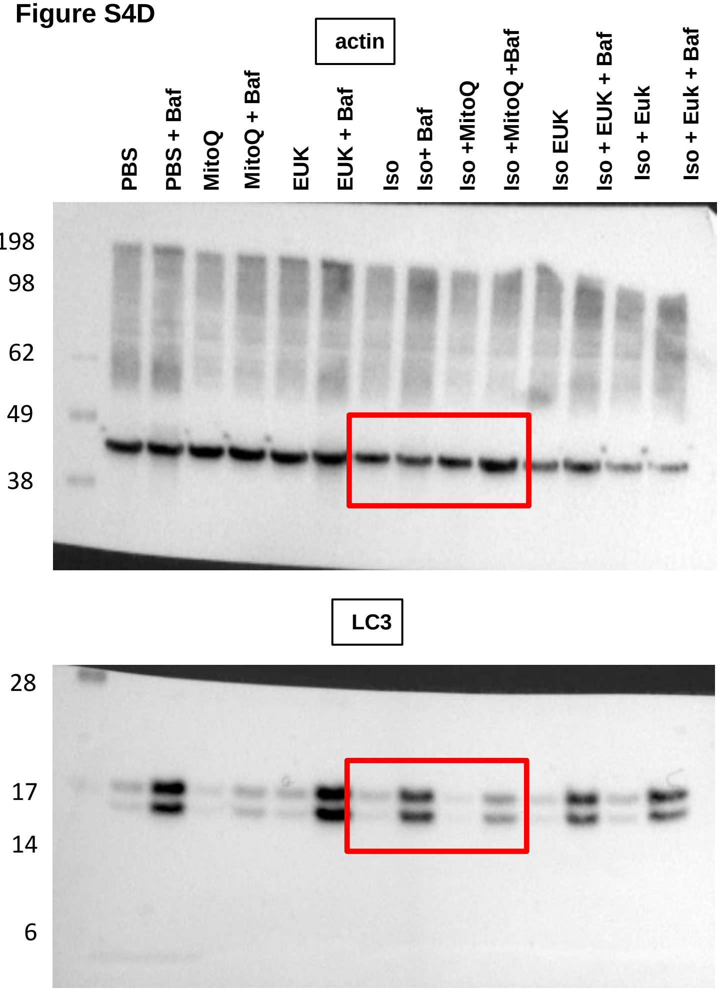

Figure S4E

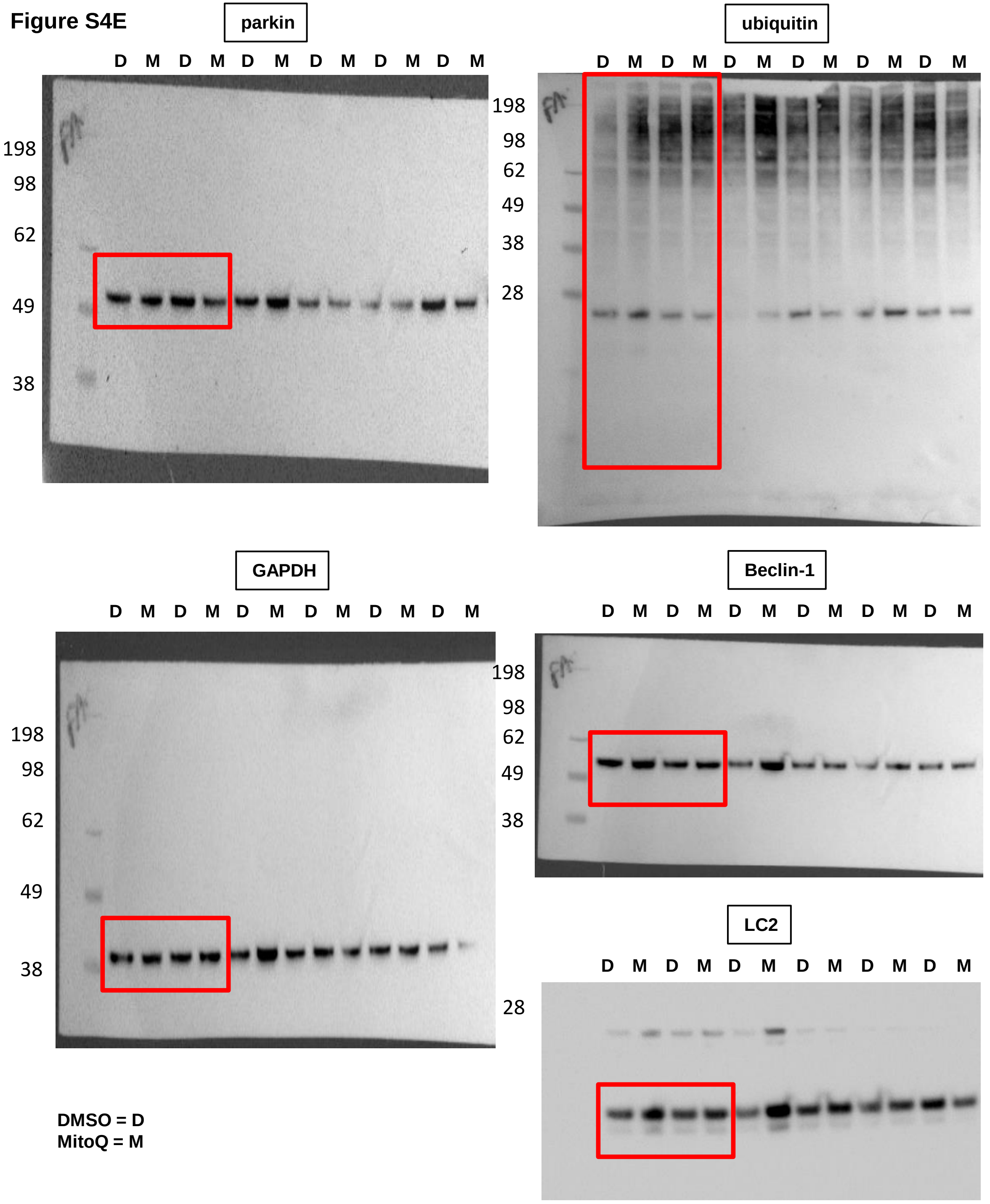

Figure 6C

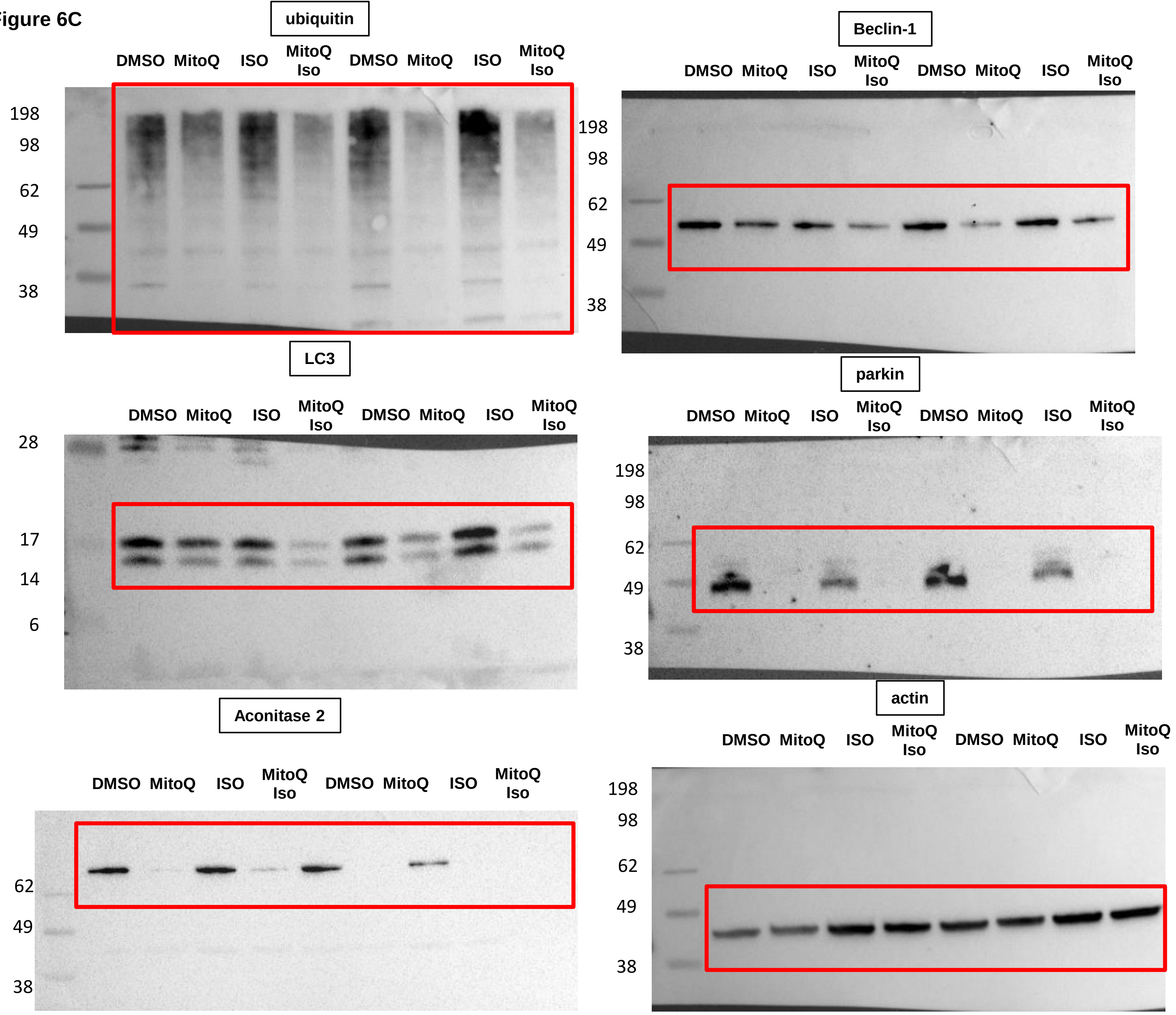
ubiquitin
DMSO MitoQ ISO MitoQ Iso DMSO MitoQ ISO MitoQ Iso
198
98
62
49
38
Beclin-1
DMSO MitoQ ISO MitoQ Iso DMSO MitoQ ISO MitoQ Iso
198
98
62
49
38
LC3
DMSO MitoQ ISO MitoQ Iso DMSO MitoQ ISO MitoQ Iso
28
17
14
6
parkin
DMSO MitoQ ISO MitoQ Iso DMSO MitoQ ISO MitoQ Iso
198
98
62
49
38
Aconitase 2
DMSO MitoQ ISO MitoQ Iso DMSO MitoQ ISO MitoQ Iso
62
49
38
actin
DMSO MitoQ ISO MitoQ Iso DMSO MitoQ ISO MitoQ Iso
198
98
62
49
38

Figure 6E

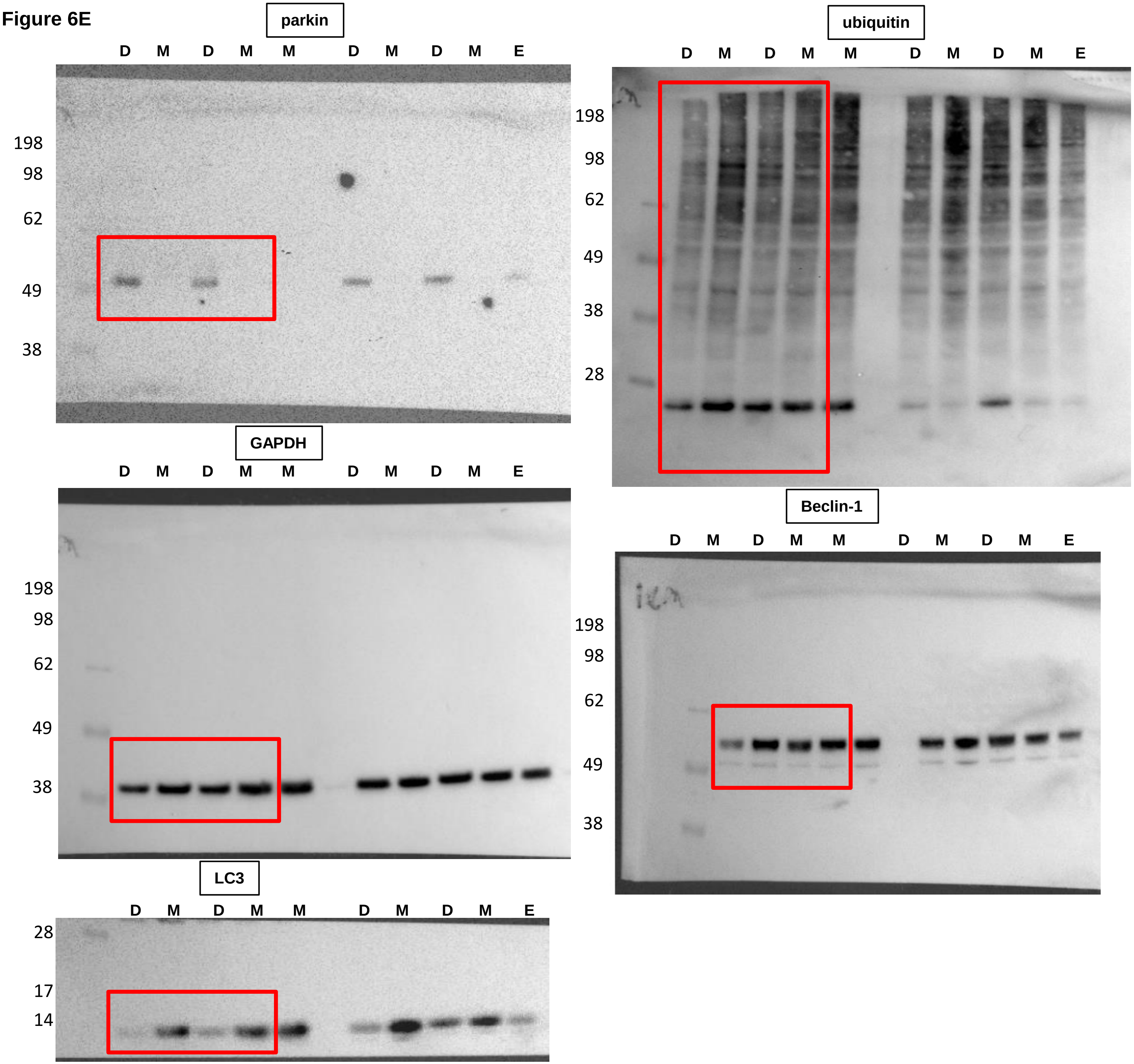

DMSO = D
MitoQ = M
EUK 134 = E

Figure 7E

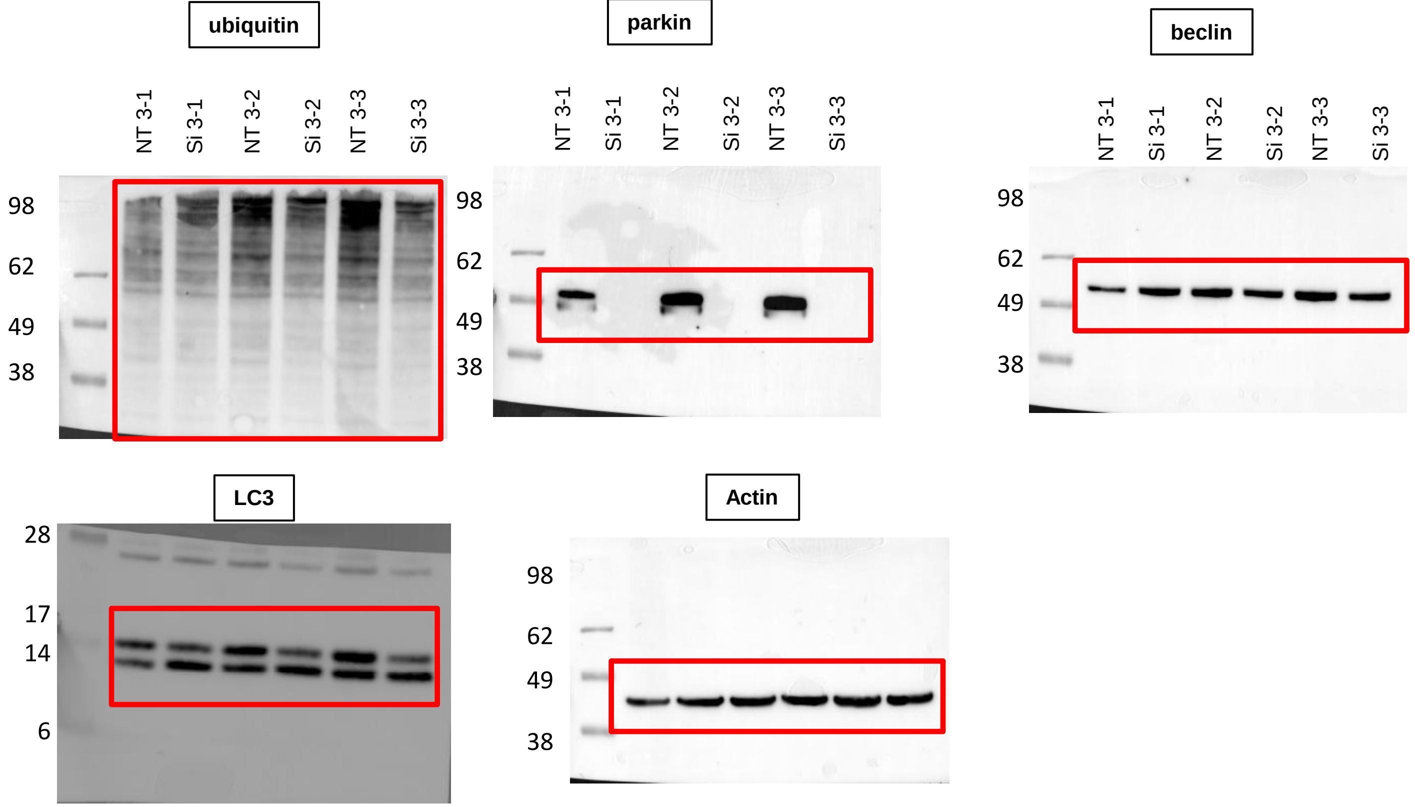
ubiquitin
NT 3-1
Si 3-1
NT 3-2
Si 3-2
NT 3-3
Si 3-3
98
62
49
38
parkin
NT 3-1
Si 3-1
NT 3-2
Si 3-2
NT 3-3
Si 3-3
98
62
49
38
beclin
NT 3-1
Si 3-1
NT 3-2
Si 3-2
NT 3-3
Si 3-3
98
62
49
38
LC3
28
17
14
6
Actin
98
62
49
38
