# Supplementary material for: Mitochondrial-Targeted Therapies Require Mitophagy to Prevent Oxidative Stress Induced by SOD2 Inactivation in Hypertrophied Cardiomyocytes
Source: Antioxidants (Basel). 2022 Apr 6;11(4):723. doi: 10.3390/antiox11040723 (PMC9029275; doi:10.3390/antiox11040723)
Supplement: Supplementary file 1 [file antioxidants-11-00723-s001.zip › antioxidants-1644144-supp-done.pdf]

Table S1: Echocardiographic, hemodynamic and morphometric parameters in sham and HF-rats.

Parameters	Sham (n=19)	2 months-HF (n=20)	*P* value
LVeDD, mm	6.6 ± 0.1	10.2 ± 0.3	<0.0001
LVeSD, mm	3.5 ± 0.2	8.6 ± 0.4	<0.0001
LVeDP, mmHg	2.02 ± 0.3	4.93 ± 1.5	0.056
LVeSP, mmHg	129 ± 5	122 ± 4	0.283
dP/dt_{max}, 10^{-3} mmHg.sec^{-1}	9.9 ± 0.4	7.9 ± 0.4	0.002
dP/dt_{min}, 10^{-3} mmHg.sec^{-1}	9.5 ± 0.6	6.6 ± 0.5	0.002
FS, %	48 ± 2	17 ± 2	<0.0001
SV, mL/beat^{-1}	0.37 ± 0.02	0.31 ± 0.02	0.016
CO, mL/min^{-1}	155 ± 8	126 ± 7	0.011
DPB, mmHg	105 ± 5	95 ± 6	0.171
BW, g	459 ± 7	462 ± 9	0.851
HW, g	1.25 ± 0.04	1.61 ± 0.08	0.0005
HW/BW, mg/g	2.74 ± 0.09	3.51 ± 0.19	0.0008
RVW, mg	211 ± 9	279 ± 23	0.009
LVW, g	0.95 ± 0.02	1.17 ± 0.05	0.0007
AW, mg	96.9 ± 8.4	160.8 ± 18.9	0.005
LW, g	1.08 ± 0.02	1.26 ± 0.12	0.158

AW, atrial weight; BW, body weight; CO, cardiac output; DPB; diastolic blood pressure; dP/dtmax, cardiac contractility; dP/dtmin, cardiac relaxation; FS, fractional shortening; HW, heart weight; LVeDD, left ventricle end diastolic diameter; LVeDP, left ventricle end diastolic pressure; LVeSD; left ventricle end systolic diameter; LVeSP, left ventricle end systolic pressure; LVW, left ventricle weight; LW, lung weight, RVW, right ventricle weight; SV, stroke volume.

Table S2: Expression of anti-oxidant and sirtuin enzymes in hypertrophied neonatal rat cardiomyocytes (NCMs).

Enzymes	PBS	Iso	*P* value
Catalase	0.94 (0.66-1.43)	0.65 (0.37-0.80)	0.073
Prx1	0.88 (0.62-1.44)	1.10 (0.64-2.81)	0.548
SOD1	1.11 (0.63-1.26)	0.92 (0.65-0.94)	0.700
SOD2	1.00 (0.57-1.43)	1.36 (0.74-1.48)	0.574
SOD2acK122 / SOD2	0.84 (0.62-1.46)	1.11 (0.57-1.25)	>0.999
SIRT1	0.84 (0.56-1.52)	0.81 (0.49-1.61)	>0.999
SIRT3	0.95 (0.68-1.35)	1.04 (0.51-1.48)	>0.999
SIRT6	0.75 (0.71-1.26)	0.85 (0.69-1.12)	0.961

Quantification of western blot. Data are expressed as median with interquartile ranges. Prx1, peroxiredoxin-1; SOD1, superoxide dismutase 1; SOD2, superoxide dismutase 2; SOD2acK122 / SOD2, SOD2 acetylated on lysine 122

Table S3: Correlation between SOD2 and sirtuins in the mitochondria by PLA.

Interaction	PBS	Iso	*P* value
SOD2 : SIRT3 (mean intensity)	18637 (804-88114)	10622 (1131-18289)	<0.0001
SOD2 : SIRT3 (number of spots)	145 (102-211)	105 (70-160)	0.0002
SOD2 : SIRT3 / mitotracker (correlation)	0.58 (0.55-0.66)	0.46 (0.42-0.48)	0.0026

Quantification of proximity ligation assay between SIRT3 and SOD2 (PLA SOD2 : SIRT3) staining in mitochondria. Mean fluorescence intensity and number of spots by cells was quantified by a "home-made" plugin on ImageJ soft- ware. Localization was quantified by pearson's coefficient, describing the correlation between PLA intensities and mitotracker images. Data are expressed as median with interquartile ranges.

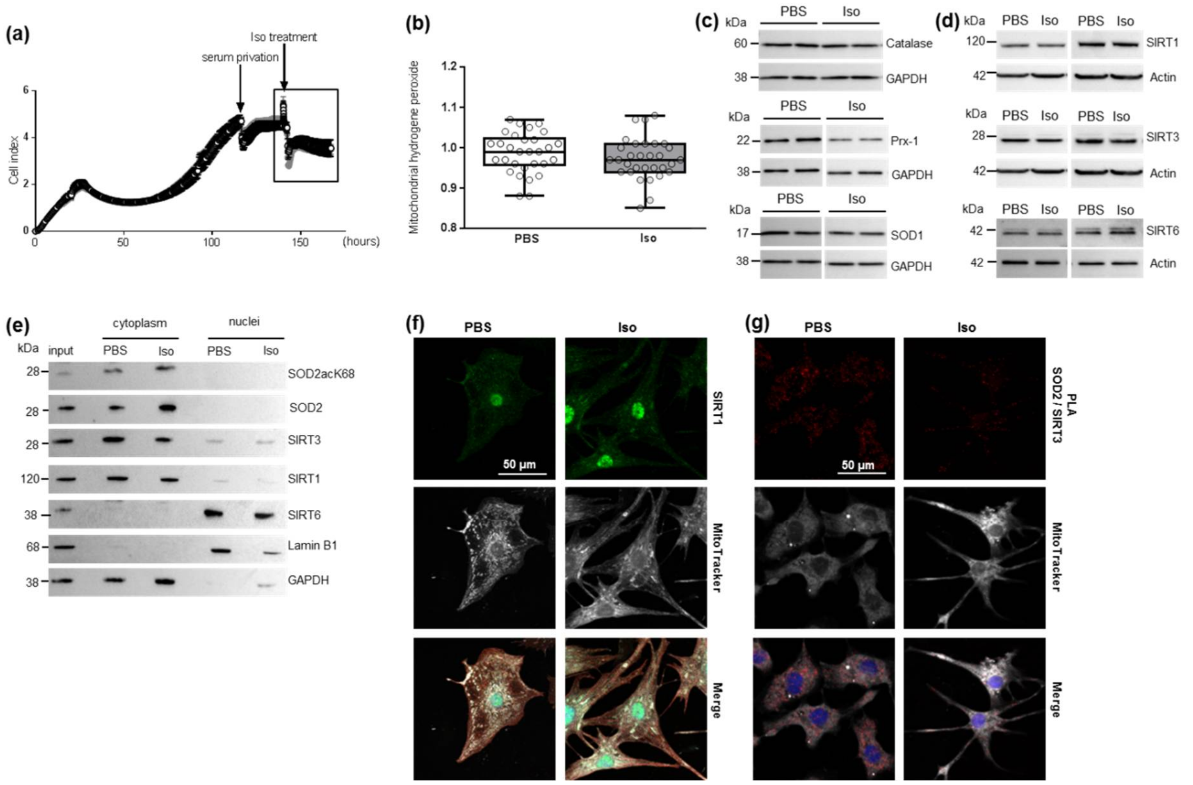

Figure S1: Characterization of mitochondrial oxidative stress in hypertrophied neonatal rat cardiomyocytes (NCMs). (**a**) Cell index quantification in untreated- (black line) and Isoproterenol (Iso) treated- (grey line) NCMs by RTCA analysis. Cell index was recorded every 15 minutes and the square represents the curve from Iso-treatment zoomed in figure 1a (n=4 independent isolation, in duplicate). Oxidative stress was also quantified in untreated (PBS) or Iso-treated NCMs for 24h by (**b**) mitochondrial hydrogen peroxide quantification and by (**c**) western blot of catalase, peroxiredoxin-1 (Prx1) and superoxide dismutase 1 (SOD1). (**c**) Western blot of SIRT1, SIRT3 and SIRT6. (**d**) Representative images of SOD2acK68 and SOD2 localized in cytoplasm after sub-cellular fractionation in untreated (PBS) and Iso-treated NCMs. (**e**) Representative images of SIRT1 (green) localized in mitochondria (white) of untreated (PBS) and Iso-treated NCMs. (**f**) Representative images of proximity ligation assay between SIRT3 and SOD2 (red) localized in mitochondria (white) in untreated (PBS) or Iso-treated NCMs for 24h. Colocalization appeared in merge images. Nuclei were stained by Dapi (blue). Data are expressed as medians with interquartile ranges for at least 3 independent experiments. Images were selected to represent the mean values of each condition.

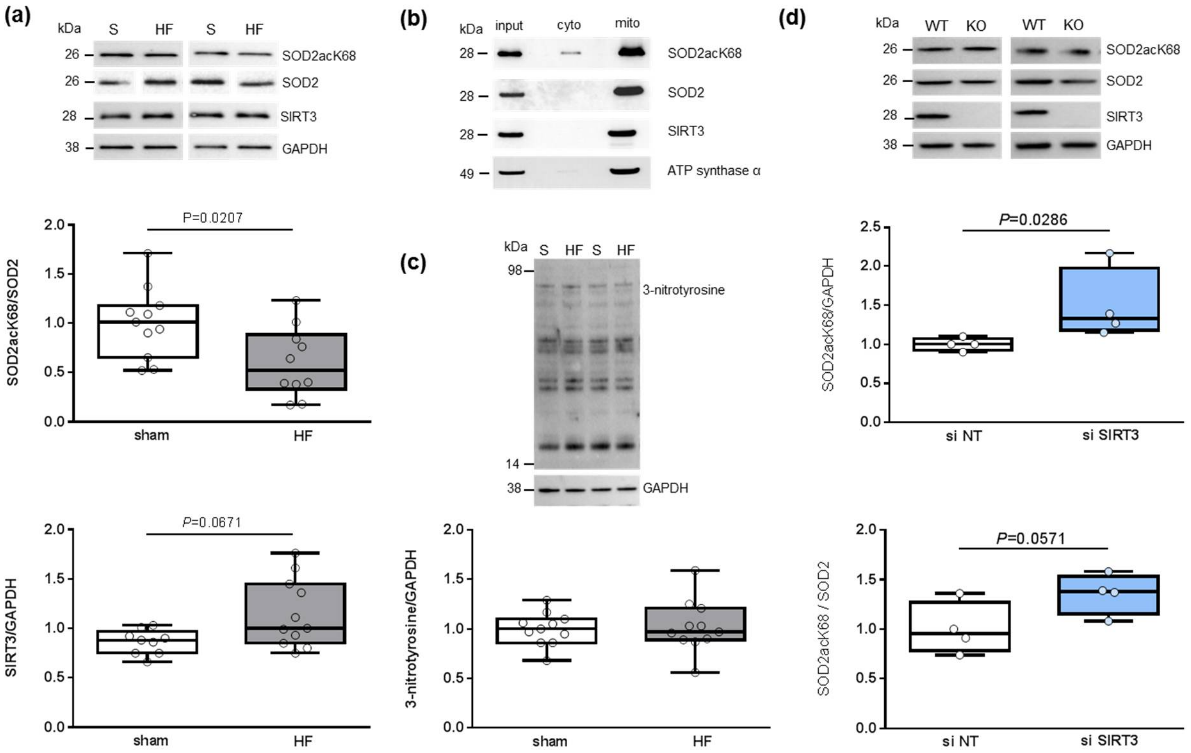

Figure S2. SIRT3 regulates SOD2 expression and its deacetylation in ischemic heart. Western blot quantification of (**a**) SOD2acK68, SOD2 and SIRT3 in LV of control- (sham) or HF-rats (HF) 2-months after MI (9 to 11 rats). (**b**) Representative images of SOD2acK68, SOD2 and SIRT3 localized in mitochondria (validated by ATP synthase α) after sub-cellular fractionation (n=4). (c) Western blot quantification of 3-nitrotyrosine in LV of the same animals. (**d**) Validation of SIRT3 inhibition and quantification of SOD2acK68 and SOD2 in isolated adult cardiomyocytes from control (WT) (n=4) and SIRT3 KO mice (n=4). Data were normalized on GAPDH. Only significant P values were indicated from at least 3 independent experiments. Images were selected to represent the mean value of each condition.

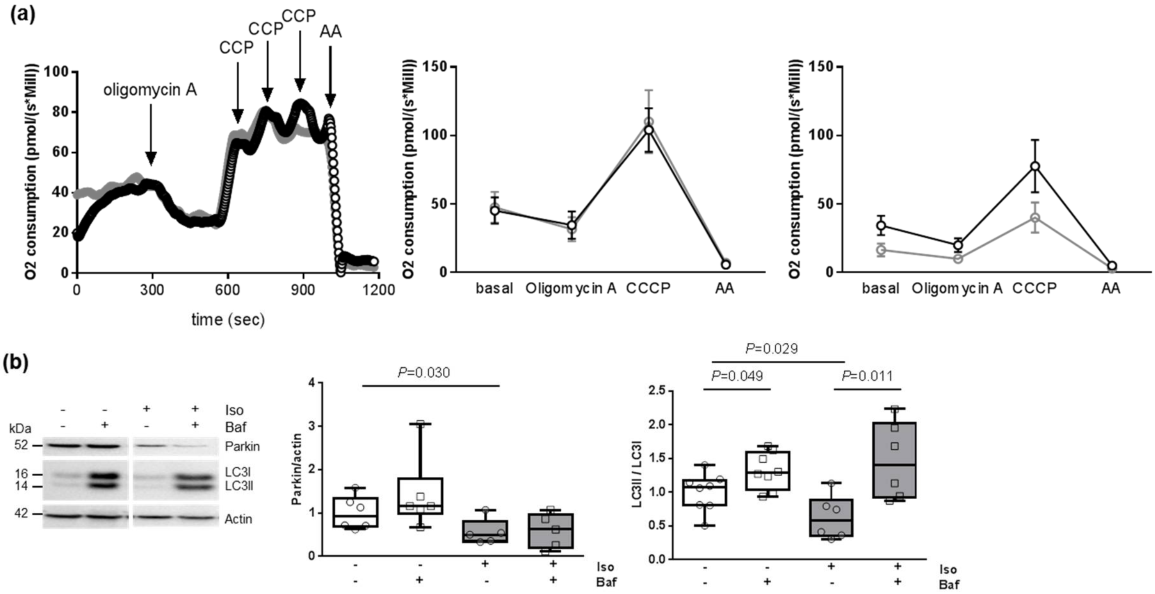

Figure S3: Characterization of mitochondrial respiration and autophagy in hypertrophied neonatal rat cardiomyocytes (NCMs). (**a**) Representative oxygen consumption at basal level and after oligomycin, carbonyl cyanide m-chlorophenyl hydrazine (CCCP) and antimycin A (AA) addition (left panel) and its quantification to characterize mitochondrial respiration (n=9) after 24h (middle panel) and 48h (right panel) in untreated- (black line) and Iso-treated (grey line) NCMs. (**b**) Mitophagy was quantified in NCMs control (-) or treated with Iso (+) with (+) or without (-) bafilomycin A1 (Baf) for 24h by western blot of parkin and LC3II/LC3I ratio. Data were normalized to actin. Only significant P values are indicated from at least 3 independent experiments. Images were selected to represent the mean values of each condition.

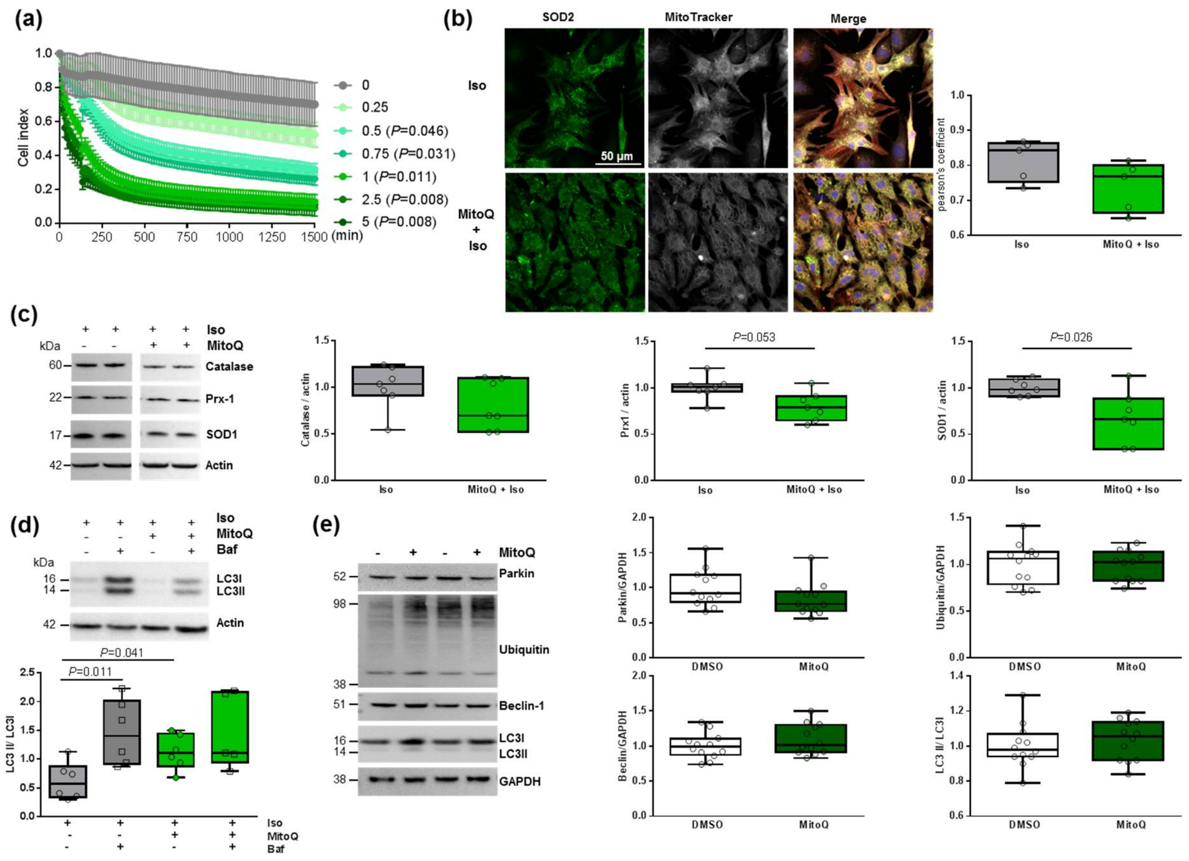

Figure S4: Effect of MitoQ in hypertrophied neonatal rat cardiomyocytes and fibroblast (NCFs). (**a**) Cell index quantification in hypertrophied NCM with pre-treatment with mitoquinone (MitoQ, from 0.25 to 5 µmol/L) (green line) or DMSO (as control) (grey line) by RTCA analysis. Cell index was recorded every 15 minutes (n=3 independent isolation). (**b**) Representative images of SOD2 (green) localized in mitochondria (white) of Iso-treated NCMs for 24h with or without MitoQ pre-treatment. Colocalization appeared in merge images. Localization of SOD2 in mitochondria was quantified by pearson's coefficient, describing the correlation between the intensities of SOD2 and mitotracker images. (**c**) Oxidative stress was quantified in Iso treated NCMs with (+) or without (-) MitoQ by western blot of catalase, peroxiredoxin-1 (Prx1) and superoxide dismutase 1 (SOD1). (d) Autophagy was quantified in NCMs treated by Iso with (+) or without (-) MitoQ pre-treatment and with (+) or without (-) Bafilomycin A1 (Baf) co-treatment by western blot of LC3II/I ratio. Data from western blot of NCMs were normalized to actin. (e) Mitophagy was quantified in NCFs treated with (+) or without (-) MitoQ pre-treatment by western blot of parkin, ubiquitinated proteins, beclin-1 and LC3II/I ratio. Data were normalized to GAPDH. Only significant P values are indicated from at least 3 independent experiments. Images were selected to represent the mean values of each condition.
